# Troponin through the looking-glass: emerging roles beyond regulation of striated muscle contraction

**DOI:** 10.18632/oncotarget.22879

**Published:** 2017-12-04

**Authors:** Jamie R. Johnston, P. Bryant Chase, Jose Renato Pinto

**Affiliations:** ^1^ Department of Biomedical Sciences, The Florida State University College of Medicine, Tallahassee, FL, 32306-4300, USA; ^2^ Department of Biological Science, The Florida State University, Tallahassee, FL, 32306-4370, USA

**Keywords:** cancer, troponin, striated muscle, nucleus, cardiomyopathy

## Abstract

Troponin is a heterotrimeric Ca^2+^-binding protein that has a well-established role in regulating striated muscle contraction. However, mounting evidence points to novel cellular functions of troponin, with profound implications in cancer, cardiomyopathy pathogenesis and skeletal muscle aging. Here, we highlight the non-canonical roles and aberrant expression patterns of troponin beyond the sarcomeric milieu. Utilizing bioinformatics tools and online databases, we also provide pathway, subcellular localization, and protein-protein/DNA interaction analyses that support a role for troponin in multiple subcellular compartments. This emerging knowledge challenges the conventional view of troponin as a sarcomere-specific protein exclusively involved in muscle contraction and may transform the way we think about sarcomeric proteins, particularly in the context of human disease and aging.

## INTRODUCTION

**Figure 1:**
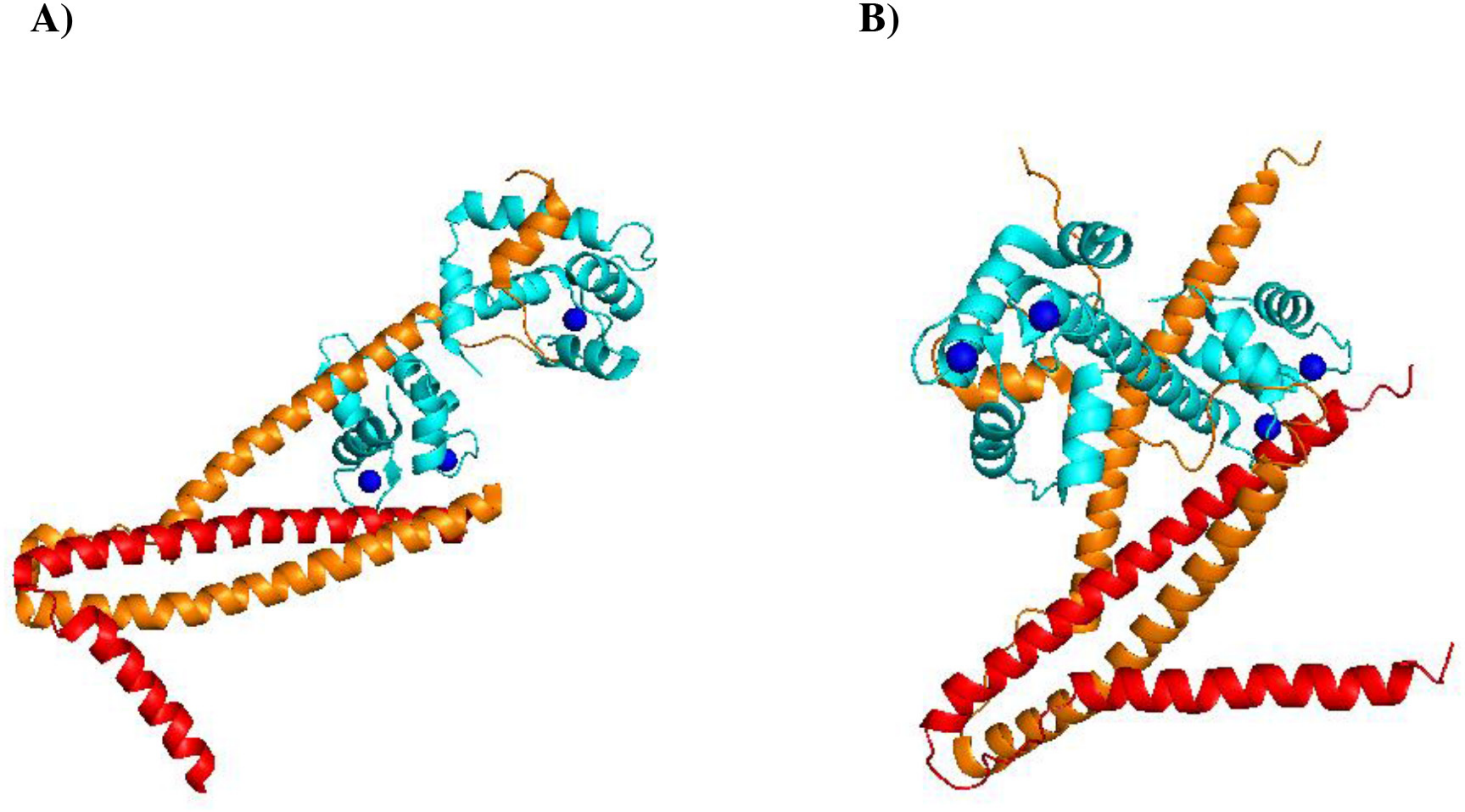
Structural representation of troponin complexes **(A)** Crystal structure of the core domain of human cardiac troponin in Ca^2+^-saturated state, PDB 1J1E. **(B)** Crystal structure of the core domain of skeletal muscle troponin in Ca^2+^-saturated state, PDB 1YTZ. Cyan: Troponin C; Orange: Troponin I; Red: Troponin T. Blue spheres: Ca^2+^ ions. Both cartoon representations were rendered using PyMol software.

Troponin is a heterotrimeric protein that is comprised of a Ca^2+^-binding subunit, troponin C (TnC), a tropomyosin-binding subunit, troponin T (TnT), and an inhibitory subunit, troponin I (TnI) [1, 2]. TnC has two known genes: one encoding a slow-skeletal/cardiac TnC isoform (*TNNC1*) and the other encoding a fast skeletal TnC isoform (*TNNC2*) [3, 4]. TnT exists as muscle fiber-specific isoforms encoded by three distinct genes: slow skeletal (*TNNT1*), cardiac (*TNNT2*), and fast skeletal (*TNNT3*) muscle; alternative splicing can further generate multiple transcript variants encoding additional TnT isoforms [5]. TnI also exists as muscle fiber-specific isoforms encoded by three distinct genes: slow skeletal (*TNNI1*), fast skeletal (*TNNI2*), and cardiac (*TNNI3*) muscle [6]. In striated muscle cells (i.e. cardiac and skeletal), troponin is situated in the thin filament, where it functions with tropomyosin (Tm) and actin to regulate muscle contraction in a Ca^2+^-dependent manner (reviewed in [7–10]). Since its discovery in the 1960s [11, 12], it has become clear that troponin plays a key role in muscle physiology. In fact, it is established that mutations in troponin subunit genes are associated with a variety of skeletal and cardiac myopathies, further underscoring the importance of this sarcomeric protein (reviewed in [13–17]). The crystal structures for both the cardiac [1] and skeletal [2] muscle troponin core domains have been solved, which have significantly advanced our understanding of the regulatory function of this sarcomeric protein in health and disease (Figure 1).

Troponin is abundantly expressed in the heart and skeletal muscle and is readily detected in myocyte thin filaments—an observation that has historically led researchers to focus primarily on understanding its role in the context of muscle contraction. However, in addition to its presence in striated muscle cells, expression of troponin genes has also been observed in tissues of the eyes [18], brain [19–22], ovaries [23], lungs [24, 25], bone [26], pancreas [27] breast [28–31], and liver [32]. Troponin mRNAs and protein subunits have also been identified in extracellular vesicles [33, 34]. Furthermore, troponin subunits have been identified in the nuclei of cardiac [35–38] and skeletal myocytes [39–41]. Interestingly, online cancer databases and functional studies have revealed that certain cancers are associated with overexpression of troponin genes. Although troponin subunits have been identified in several subcellular compartments across multiple cell types, functional roles beyond striated muscle contraction are only beginning to emerge.

It is perhaps not surprising that troponin subunits have been identified in subcellular compartments beyond the sarcomeric apparatus. Indeed, despite initial controversy, it is now undisputed that actin and motor proteins classically associated with the cytoskeleton have functions in the nuclear compartment. Specifically, nuclear actin [42–49] and myosin I [50–55] play key roles in transcriptional regulation and chromatin remodeling (reviewed in [56, 57]). Tropomyosin, a troponin-binding partner in striated muscle thin filaments, has also been identified in nuclei of various cell types [35, 58, 59]. Moreover, a specific domain of titin, another protein associated with the sarcomere in muscle cells, has been shown to localize to the nucleus and regulate cell proliferation [60]. While the novel functions of these cytoskeleton/sarcomere-associated proteins in the nucleus are described elsewhere, the non-canonical functions of troponin have not been previously summarized. In this review article, we provide a comprehensive overview of literature concerning the atypical, cell-specific expression patterns and multifaceted functions of troponin beyond muscle contraction—with an emphasis on its roles in cancer, cardiomyopathy pathogenesis, and skeletal muscle aging. We also utilize multiple bioinformatics approaches to invoke potential mechanistic insight and ultimately offer an unconventional perspective on troponin.

## TROPONIN IS ABERRANTLY EXPRESSED IN CANCER

According to multiple online cancer databases and inchoate functional studies, it is becoming clear that troponin subunits, at the level of gene and protein expression, are aberrantly expressed in specific, often metastatic, forms of cancer. In the setting of cancer, troponin subunits seem to be regulating diverse cellular processes related to tumor growth and metastasis. Interestingly, somatic mutations in troponin genes are also associated with cancer, and evidence suggests that certain troponin genes may function as proto-oncogenes.

### Cancer databases reveal overexpression of troponin genes

A significant proportion of certain cancers documented in online databases exhibit over-expression of genes encoding troponin I isoforms [25]. Further analysis of the online catalogue of somatic mutations in cancer (COSMIC, http://cancer.sanger.ac.uk/cosmic) database also reveals altered expression, by gene amplification or expression changes, of other troponin genes. Specifically, COSMIC shows over-expression of fsTnI (*TNNI2*, Id: COSG67231) and cTnI (*TNNI3*, Id: COSG75182) genes. Consistent with this finding, immunohistochemistry and immunofluorescence studies showed aberrant expression of cTnI protein in human non-small cell lung cancer tissue and cancer cell lines [24], as well as in endothelial cells of the rat brain [61]. Furthermore, the genes encoding cTnC (*TNNC1;* Id: COSG56773), fsTnC (*TNNC2*; Id: COSG65631), and all three TnT isoforms (*TNNT1*, Id: COSG65275; *TNNT2*, Id: COSG67007; *TNNT3*, Id: COSG60869) are overexpressed in several cancer types according to the COSMIC database. It should also be noted here that TnT’s binding partner in striated muscle cells, e.g., α-Tm (*TPM3*), is also overexpressed in cancers of the breast, endometrium, cervix, liver, and esophagus. A notable pattern emerges upon further inspection of the cancer type and affected tissue with altered troponin gene expression: overexpression of the troponin genes appears to be closely associated with cancers of the biliary tract, large intestines, pancreas, endometrium, lung, ovary, stomach, esophagus, thyroid and skin. For instance, approximately 40% (238 out of 610) of tissue samples from cancer of the large intestines show overexpression of *TNNC2* in the COSMIC database. Specifically, there is a trend toward carcinomas (epithelial cell origin) and melanoma cancer types. Of the three TnI gene isoforms, *TNNI1* shows the highest overexpression in cancers of the brain (i.e. glioma and medulloblastoma). Elucidating the functional relevance of *TNNI1* overexpression in aggressive brain tumors may provide a platform for developing novel therapies and diagnostic markers, and ultimately advance our understanding of tumor pathogenesis in the brain.

Utilizing a second cancer database, (http://www.cbioportal.org), a similar pattern of altered troponin gene expression is observed. The alteration frequency is based on amplification, deletion, or mutation of the troponin genes, as described on the cBioPortal [62, 63]. Altered expression of *TNNC1* (primarily by gene amplification) is associated with adenocarcinoma of the prostate, pancreas, and esophagus, as well as carcinoma of the breast, kidneys, and bladder in a proportion samples examined [62, 63]. A similar trend is observed for the remaining troponin genes. For instance, amplification of *TNNT2* is associated with neuroendocrine prostate cancer and breast cancer in a certain proportion of patient samples [62, 63].

It is important to note that both the COSMIC and cBioPortal databases reveal an interesting phenomenon: mutations in all eight troponin genes are found in certain human cancers. Somatic mutations for the troponin genes obtained from the COSMIC database are shown in Supplementary Tables 1-8. Notably, missense mutations predominate other forms of mutations (Supplementary Tables 1-8). We recognize that silent (synonymous) mutations could have an effect in cancer [64], but they have been omitted for brevity. Although it is well known that inherited and *de novo* mutations in troponin cause various forms of cardiomyopathy [15], it is not clear whether the somatic mutations observed in cancer cell genomes have any functional relevance. Examining the genomic location of the mutations in troponin genes associated with certain cancers may provide insight into the potential function of these mutations in the context of tumor biology. It should be acknowledged that mutations in troponin genes could be a consequence, rather than an initiator, of tumor development. Finally, it is critical to point out that the altered troponin gene expression is associated with a certain proportion of samples tested and is not a trend observed across all tumor samples tested. Tumor heterogeneity and differences in staging are two factors that could contribute to the proportions of samples with *vs.* without altered troponin expression. Nevertheless, there is a remarkable association between mutations in troponin genes and certain cancers—a novel finding that warrants scientific inquiry.

### Troponin in cancer cells: evidence at the protein level

**Figure 2:**
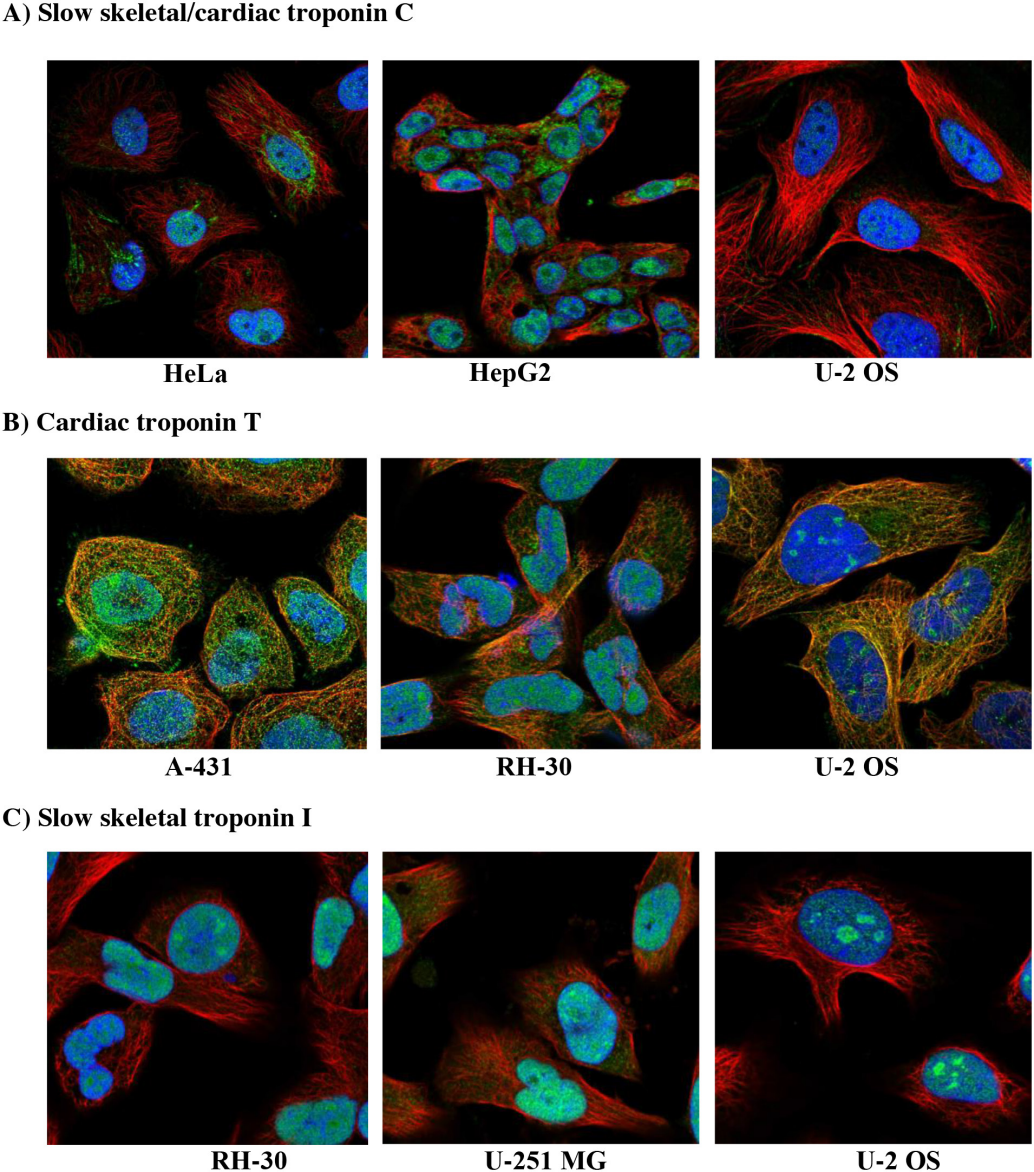
Immunocytochemistry images of troponin in human cancer cell lines **(A)** Slow skeletal/cardiac troponin C staining in cervical carcinoma (HeLa), liver carcinoma (HepG2) and osteosarcoma cells (U-2 OS). **(B)** Cardiac troponin T staining in epidermoid carcinoma (A-431), rhabdomyosarcoma (RH-30), and U-2 OS cells. **(C)** Slow skeletal troponin I staining in RH-30, glioblastoma (U-251) and U-2 OS cells. In all cases, green represents the troponin subunit, blue staining is the nucleus and red is microtubules. Images obtained from (http://www.proteinatlas.org/cancer).

In addition to the patterns of altered troponin gene expression curated in online cancer databases, expression of troponin is also borne out by evidence at the protein level. Although it is possible that troponin mRNA can be expressed in the cell without translation to protein, analysis of immunostaining images and proteomic data from online databases confirm the presence of troponin protein in cancer cells. Immunocytochemistry images obtained from the cancer atlas on The Human Protein Atlas database (https://www.proteinatlas.org) show clear evidence of troponin protein expression in multiple human cancer cell lines [65]. For instance, immunostaining for cTnC is positive in the nucleoplasm of cervical carcinoma cells (HeLa), hepatocellular carcinoma cells (HepG2), and osteosarcoma cells (U-2 OS) (Figure 2A). Positive staining for cTnC is associated not only with the nucleoplasm of all three cell types, but also the mitochondria of HeLa and HepG2s [65]. TnC had not previously been observed in mitochondria and is a finding that warrants further investigation. Positive staining for the cTnT isoform is observed in epidermoid carcinoma (A-431), rhabdomyosarcoma (RH-30), and U-2 OS cells (Figure 2B). cTnT staining is associated with the nucleus and nucleolus in all three cancer cell types, and focal adhesion sites in A-431 and U-S OS cells. The existence of cTnT at focal adhesion sites raises the possibility of this troponin subunit being involved in cell motility and division, two processes that are typically upregulated in malignant tumors. Finally, RH-30, glioblastoma (U-251) and U-2 OS cells demonstrate positive staining for ssTnI in the nucleoplasm (Figure 2C). Interestingly, it appears ssTnI is enriched in the dense nucleolar regions in all three cancer cell types, suggesting it may participate in transcription and processing of rRNA genes. Since cancer cells are highly metabolic and require rapid protein synthesis, this is a tenable supposition. Based on these observations, it seems that troponin subunits in cancer cells exhibit subcellular compartment specificity, suggesting they may function in diverse cellular processes.

Global, high throughput mass spectrometry data curated on PhosphoSitePlus (http://www.phosphosite.org) support the presence of troponin subunits, post-translationally modified, in human cancer cells and tissue. For instance, in a study using phospho-tyrosine immunoaffinity beads, cTnC (P63316) was found to be phosphorylated at tyrosine 111 (TnC-Y111-P) in human gastrointestinal cancer tissue, non-small cell lung cancer tissue, B cell lymphoma cell line (OCI-ly18), non-small cell lung cancer cells (NCI-H1650), and pancreatic carcinoma cells (PANC-1) [66]. Furthermore, dimethylated arginine residues and an acetylated-lysine residue were detected on fsTnI (P48788) in human colorectal carcinoma cells [66]. The functional relevance of these modified residues remains unclear, but these modifications seem to be consistent with the regulation of nuclear proteins. In a study evaluating the impact of ischemia on global changes in phosphorylation of proteins in human ovarian and breast tumor tissue, multiple troponin subunits were identified by tandem mass spectrometry [67]. Specifically, cTnC and cTnI were identified in high-grade serous ovarian cancer, a malignancy that accounts for approximately 75% of human ovarian cancer deaths [68]. In the human breast cancer tumors, fsTnT, ssTnT, fsTnI, ssTnI, fsTnC, and cTnC were all identified [67]. The presence of all three subunits in the breast cancer tissue suggests that troponin may exist as a complex rather than individual subunits. The gene and protein expression data from online cancer databases provide compelling support for the aberrant expression of this sarcomeric protein in certain cancers.

### *TNNC1*: identification of a proto-oncogene?

Several studies have explored the role of TnC in the context of tumor growth and metastasis. Insertional mutagenesis experiments [69] in rodent models demonstrated that the cTnC gene drives the progression of intestinal tumorigenesis [70] and hepatocellular carcinoma in a hepatitis B mouse model [71]. Moreover, as already noted, mutations in the *TNNC1* gene are associated with certain cancers in the COSMIC database. Taken together, these findings support a role for *TNNC1* as a potential (proto-) oncogene. A microarray expression analysis using a SW480 colon cancer cell line revealed that *TNNC1* is differentially expressed depending on the activation of different chemokine receptors (CXCR4 and CXCR7) [72]. These microarray results were further validated with quantitative polymerase chain reaction (qPCR), specifically examining transcription of *TNNC1*, among others [72]. While changes in gene expression are not always commensurate with alterations in protein expression, cTnC has been observed at the protein level in human lung adenocarcinoma cells (SPCA-1) and human gastric adenocarcinoma cells (BGC 823) [24]. Changes in *TNNC1* expression were also demonstrated in human tongue squamous cell carcinoma (TSCC) tissue with concomitant cervical lymph node metastasis (CLNM) [73]. Gene microarray analysis and immunohistochemistry results indicated altered *TNNC1* expression in early-stage human oral cancer and its association with poor survival [73]. Specifically, the results showed a strong association between *TNNC1* expression (among others) and CLNM [73]. It was also noted that *TNNC1* might ultimately serve as a future clinical prognostic marker for TSCC and its occult cervical lymphatic metastasis [73].

Ca^2+^ signaling is known to play an important role in cancer [74–76]. Given the inherent Ca^2+^-binding properties of TnC, a link between calcium signaling and TnC in cancer cells is conceivable. The first glimpse of mechanistic insight into the role of TnC in cancer emanated from a study on the metastatic potential of human ovarian cancer. Specifically, it was shown that *TNNC1* is overexpressed in epithelial ovarian cancer cells, as well as human high-grade serous ovarian cancer tissue [23]. Furthermore, siRNA- knockdown of *TNNC1* abrogated the stimulatory effect of microfibrillar-associated protein 5 (MFAP5) on ovarian cancer cell motility, indicating *TNNC1* as a downstream effector of MFAP5 [23]. Treatment of the ovarian cancer cells with recombinant MFAP5 and subsequent immunostaining showed increased density and organization of the F-actin cytoskeleton, which was abrogated by siRNA knockdown of *TNNC1*. Therefore, it was proposed that *TNNC1* likely functions mechanistically by facilitating the formation and reorganization of cytoskeletal F-actin to enhance traction forces that underlie motility of ovarian cancer cells. In conclusion, a Ca^2+^-dependent signaling complex (FAK/CREB/TNNC1) was implicated in fostering the metastatic potential of ovarian cancer [23]. It is important to note, however, that this study did not confirm protein expression of *TNNC1* and that modulating the gene was sufficient for the observed signaling effects. Finally, it was shown that RNA-mediated knockdown of *FOXE1*, a gene located at a chromosomal locus associated with risk of papillary thyroid cancer, results in up-regulation of *TNNC1* gene expression in human thyroid cells [77]. Further studies are needed to clarify the molecular mechanisms by which *TNNC1* contributes to malignant cell growth and should include experiments that determine whether expression of TnC protein is required for the observed effects.

### Fast skeletal TnI acts as a tumor suppressor

Angiogenesis is a crucial component of solid tumor growth and metastasis, as the new blood vessels provide a route for delivery of nutrients, removal of wastes, and transport of metastatic cancer cells to distant parts of the body [78–80]. The discovery of fsTnI in cartilage and its potent inhibitory effects on blood vessel growth [81–83] has prompted further investigation into its potential to suppress tumor growth. For example, it was previously shown that extracellular TnI could inhibit basic fibroblast growth factor (bFGF)–stimulated endothelial cell proliferation [84]. Based on the *in vitro* results, it was proposed that fsTnI may be acting, in part, via interactions with the cell surface receptor, bFGF [84]. Interestingly, fsTnI exerted its anti-proliferative effects on non-endothelial cells as well [84]. It has also been proposed that fsTnI may inhibit endothelial cell proliferation via heparin binding to compete for the bFGF receptor [81]. These studies provide support for fsTnI functioning outside of the cell via interactions with an extracellular receptor, which contrasts intracellular functions of other TnI isoforms. The anti-angiogenic property of fsTnI and its inhibitory effects on endothelial cell proliferation extend beyond cell culture studies. Using a peptide containing the inhibitory region of human fsTnI (Glu94-Leu123), it was demonstrated *in vivo* that this peptide could inhibit pancreatic cancer metastasis in a liver metastases mouse model [27]. This study not only confirmed the ability of fsTnI to inhibit tumor metastasis in a mammal, but it also determined a region of fsTnI responsible for the anti-tumor effects. This does not, however, exclude the possibility of other functionally relevant regions in fsTnI.

Another *in vivo* study reported that expression of fsTnI thwarted the growth of a primary ovarian carcinoma in mice by inhibiting angiogenesis [85]. fsTnI seems to modulate tumor growth by multiple mechanisms. For example, it was demonstrated *in vivo* that overexpression of fsTnI in hepatoma cells inhibits tumorigenesis, perfusion, and vascularization, marked by induction of apoptosis and a reduction in cell proliferation [86]. A subsequent study revealed that transfer of the fsTnI gene into a rat hepatoma resulted in global gene expression changes as well as fluctuations in glucose metabolism [87]. The inhibitory region of fsTnI corresponds to an actin-binding region in the myofilament [6, 88, 89]. Therefore, another potential mechanism by which fsTnI inhibits endothelial cell growth is perhaps by saturating the myosin-binding sites on F-actin, thus impeding cytoplasmic actin dynamics required for mitosis and cytokinesis. This proposed mechanism may involve the presence of Tm to facilitate fsTnI-actin interactions as in striated muscle regulation, but remains ambiguous since it is also known that TnI can bind to actin in the absence of Tm. Alternatively, it could also interfere with nuclear acto-myosin-mediated transcription of genes associated with cell growth [57]. Taken together, fsTnI likely elicits its effects via multiple molecular mechanisms. Although the *in vivo* effects of TnI seem to be clear, one study failed to demonstrate therapeutic efficacy using TnI gene therapy in a transplantable osteosarcoma rat model, despite convincing findings on its inhibition of endothelial cell growth *in vitro* [90]. As addressed by the authors, the inconsistent findings could be due, in part, to the susceptibility of the secreted form of fsTnI to degradation *in vivo* via matrix metalloproteinases [90]. Nevertheless, evidence from multiple studies support a role for fsTnI in regulating endothelial cell growth and angiogenesis.

### Slow skeletal TnI promotes tumorigenesis

The ssTnI isoform has also been implicated in tumor growth, but the effects of ssTnI on tumorigenesis appear to diametrically oppose those of fsTnI. It was previously reported that TnI regulates chromosomal stability and cell polarity in early *Drosophila* development [58]. Based on the prior study and observation of *TNNI1* overexpression in cancer databases, the same group explored the potential link between ssTnI expression and cancer in a subsequent investigation. Interestingly, ssTnI was shown to localize in the nuclear compartment and up-regulate genes involved in cell growth in *Drosophila* S2 cells [25]. In another study, down-regulation of the *TNNI1* gene quelled proliferation of human non-small-cell lung carcinoma xenografts in mice [25]. Overall, this study implicated ssTnI’s involvement in tumor growth. The cardiac isoform of TnI has been identified in human non-small cell lung carcinoma tissue by immunohistochemical staining, though its functional relevance remains unclear [24].

Remarkably, ssTnI and fsTnI appear to elicit opposing effects in terms of cell growth. Perhaps fsTnI acts a tumor suppressor gene, while ssTnI promotes tumor growth by functioning as an oncogene. This supposition seems reasonable since mutations in *TNNI1* and *TNNI2* are found in human cancer reported in online databases (Supplementary Tables 3 and 4). Since it was previously shown that knockdown of *TNNI1* in a mouse model bearing non-small-cell lung carcinoma xenografts restrained cell proliferation [25], it would be worthwhile to determine if targeted knockdown of *TNNI1* in certain cancers thwarts tumor growth and metastasis in larger animals models. Targeting TnI in an isoform-specific manner in the tumor microenvironment of cancers expressing this subunit may be indicated, since this protein is not highly expressed in healthy, non-striated muscle cells. Nonetheless, it is clear that elucidating the isoform-dependent differences of TnI in malignant cell growth would certainly broaden our understanding of this troponin subunit found in certain cancers. Moreover, a mechanistic understanding of TnI specifically in the context of tumor metastasis could pave the way for the development of targeted therapy in patients with certain malignant cancers.

### Expression of TnT isoforms in cancer

Functional studies focusing on TnT isoforms in cancer are lacking, although one report suggests that the ssTnT gene may play a role in cancer cell immortalization [91]. In this study, it was found that human induced pluripotent stem cells (hiPSCs) and immortalized retinal pigment epithelial (RPE) cells exhibited a 100-fold increase in *TNNT1* expression compared to primary RPE cells. Also, this group used a TissueScan array technique and detected significant overexpression of *TNNT1* in human cancers of the cervix, colon, lung, ovary, and testis compared to normal tissue [91]. A remarkable finding from this study demonstrated that overexpression of *TNNT1* in retinal pigment epithelial cells stabilized actin filaments and augmented cell migration [91]. This is the first study to implicate a functional role for *TNNT1* in cancer-related processes. Mechanistically, it was suggested that TnT likely functions with Tm (its cognate binding partner) to enhance cell migration [92]. The potential involvement of Tm in these proposed mechanisms is consistent with the findings of *TPM3* overexpression in online cancer databases. A separate study examining gene expression changes in human endometrial carcinoma tissue revealed that *TNNT1* is differentially expressed, depending on the specific subtype of endometrial carcinoma [93].

Altered expression of the three TnT genes documented in the online cancer databases also extends to the protein level. The Human Protein Atlas database (https://www.proteinatlas.org) contains isoform-specific evidence of TnT immunostaining in various human cancer tissues. A subset of tumor cells in papillary adenocarcinoma of thyroid tissue exhibited positive staining for cTnT, as did a case of colon cancer [65]. On the other hand, cases of melanoma, urothelial, and testicular cancer displayed positive staining for fsTnT. Interestingly, staining for ssTnT was negative in the cancer tissues tested, which is inconsistent with gene expression analysis performed in a previous study [93]. Although variations in sample type and preparation may account for this disparity, it is also possible that *TNNT1* is (post)-transcriptionally regulated or has a short protein half-life. Expression of cTnT protein has also been observed in a human tissue sample from a patient with renal cell carcinoma [94]. Furthermore, a positive correlation between *TNNT1* expression and human gallbladder carcinoma tumor progression was recently reported [95].

**Figure 3:**
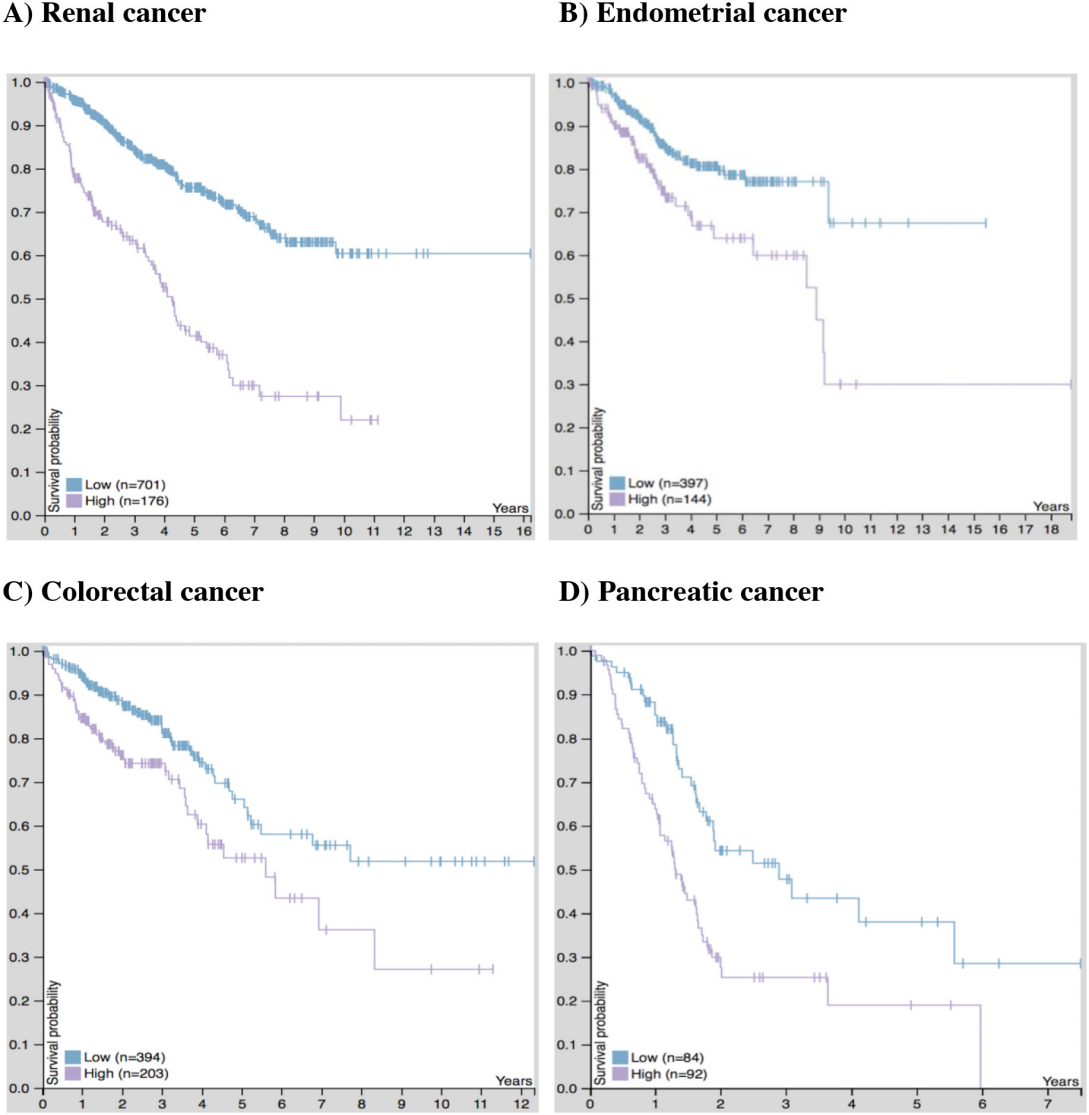
Kaplan-Meier plots for *TNNT1* expression in human cancers “High” expression of *TNNT1* in **(A)** Renal **(B)** Endometrial **(C)** Colorectal and **(D)** Pancreatic cancer is associated with an unfavorable (poor survival) prognosis. Plots represent the best separation between high (purple tracing) and low (blue tracing) mRNA expression based on fragments per kilobase million (FPKM) cut-off values that provided the highest level of significance. The FPKM cut-off values for renal, endometrial, colorectal, and pancreatic cancer were 0.5, 17.5, 0.7, and 1.1, respectively. Plots and calculated values were obtained from the online Human Pathology Atlas database on August 28^th^, 2017 [96].

To better understand the link between troponin gene expression and patient survival, we included a Kaplan-Meier analysis related to *TNNT1* expression and cancer obtained from The Human Protein Atlas (http://www.proteinatlas.org/pathology) [96]. Patients with renal, endometrial, colorectal, and pancreatic cancer concomitant with high *TNNT1* expression have an unfavorable prognosis compared to patients with low *TNNT1* expression (Figure 3). This trend is most pronounced in pancreatic cancer patients with high *TNNT1* expression, where survival probability drops precipitously within a 2-year time span (Figure 3D). Considering the advances regarding functional roles for TnC and TnI, it seems likely that TnT is also involved in cancer cell growth and migration. Functional studies are needed to clarify the potential role(s) of TnT isoforms in cancer development.

### A cardiac-specific TnI-interacting kinase is aberrantly expressed in tumors

Kinases are a class of enzymes that represent central targets for treating various forms of cancer [97]. *TNNI3K* is a gene that encodes a human cardiac troponin I interacting (serine/threonine) kinase, which belongs to the MAPKKK family [98]. It was previously reported that expression of this gene is restricted to cardiac muscle in healthy individuals and is important for physiological heart growth [98, 99]. The importance of *TNNI3K* has primarily been studied in the setting of heart disease, where it has been shown to drive progression of various cardiomyopathies [100]. Interestingly, the COSMIC database and cBioPortal show that this gene is abnormally expressed in a proportion of patients with cancers such as adrenocortical, breast, and carcinomas, as well as acute myeloid leukemia [62, 63]. Since its substrate, troponin I, is aberrantly expressed in multiple cancer types at the gene and protein levels, perhaps the activity of TnI is regulated by this TnI-interacting kinase in cancer cells. Given its known roles in promoting physiological and pathological growth of the heart, it may have a similar role in cancer cells. A selective inhibitor of TnI-interacting kinase 3 exists [101] and may be useful for dissecting potential *TNNI3K*-dependent signaling pathways in cancer cells. It was previously shown that cTnI protein is abnormally expressed in human non-small cell lung carcinoma tissue and human lung cancer cell lines [24]; hence, focusing efforts on the potential role of this kinase in lung cancer may be a judicious starting point. Further studies should aim to confirm the expression of *TNNI3K* at the protein level in human cancer cells, determine the subcellular localization of this kinase, and ascertain whether it may play a role in malignant cell growth. Finally, since targeting kinases is an established therapeutic strategy for treating various forms of cancer, understanding the potential functional role of this TnI-interacting kinase in the context of tumorigenesis represents an important step towards potentially modulating the activity of this enzyme for anti-tumor therapy.

### Troponin subunits are present in cancer cell-derived extracellular vesicles

Extracellular vesicles (EVs) are membranous structures primarily involved in intercellular communication [102]. These signaling pods can be of endosomal or plasma membrane origin, and are referred to as exosomes and microvesicles, respectively [103]. Growing evidence has implicated EVs in growth, invasion, and metastasis of cancer cells [104, 105]. Analysis of published transcriptomics and proteomics studies on multiple human cancer cell lines has uncovered the unusual presence of troponin messenger RNAs (mRNAs) and protein subunits in extracellular vesicles. A global transcriptomics analysis on microvesicles derived from human colorectal cancer cells (CRCs) found that EVs were enriched with cell-cycle related mRNAs [34]. Among these microvesicular mRNAs were *TNNC1, TNNI3*, and *TNNT1* transcripts. A remarkable finding from this study also showed that the CRC-derived microvesicles stimulated proliferation of endothelial cells, suggesting that colorectal tumor growth and metastasis may be mediated by microvesicles transporting angiogenesis-related molecules. These findings suggest the possibility that troponin may contribute to tumor growth and metastasis via EVs.

Although it was previously demonstrated that fsTnI inhibits endothelial cell proliferation by interacting with an extracellular receptor [84], the role of the cardiac isoform present in CRC-derived microvesicles is unknown. Nevertheless, the two isoforms could have distinct functions in cancer. In a separate study on patient-derived, primary cultures of glioblastoma cells, microarray analysis identified the presence of *TNNT1* mRNA transcripts in secreted microvesicles [106]. In addition to troponin mRNAs being identified in EVs, proteomics studies have confirmed the presence of troponin subunits at the protein level. A comprehensive proteomic profiling of extracellular vesicles from NCI-60 human cancer cells has been published [33]. Among the 60 cancer cells examined, troponin subunits were identified by liquid chromatography tandem mass spectrometry (LC-MS/MS) in EVs derived from two human cancer cell lines: cTnC in non-small cell lung carcinoma cells (NCI-H522), and fsTnT in malignant melanoma cells (NCI-LOX-IMVI). It is worth noting that these findings are consistent with the evolving theme of troponin’s association with metastasis-related processes in cancer. By highlighting troponin-related findings from global analyses on EV cargo, we hope to provide the impetus for future investigations to assess the functional significance of this protein in cancer cell-derived EVs.

### Outlook on troponin in cancer

**Figure 4:**
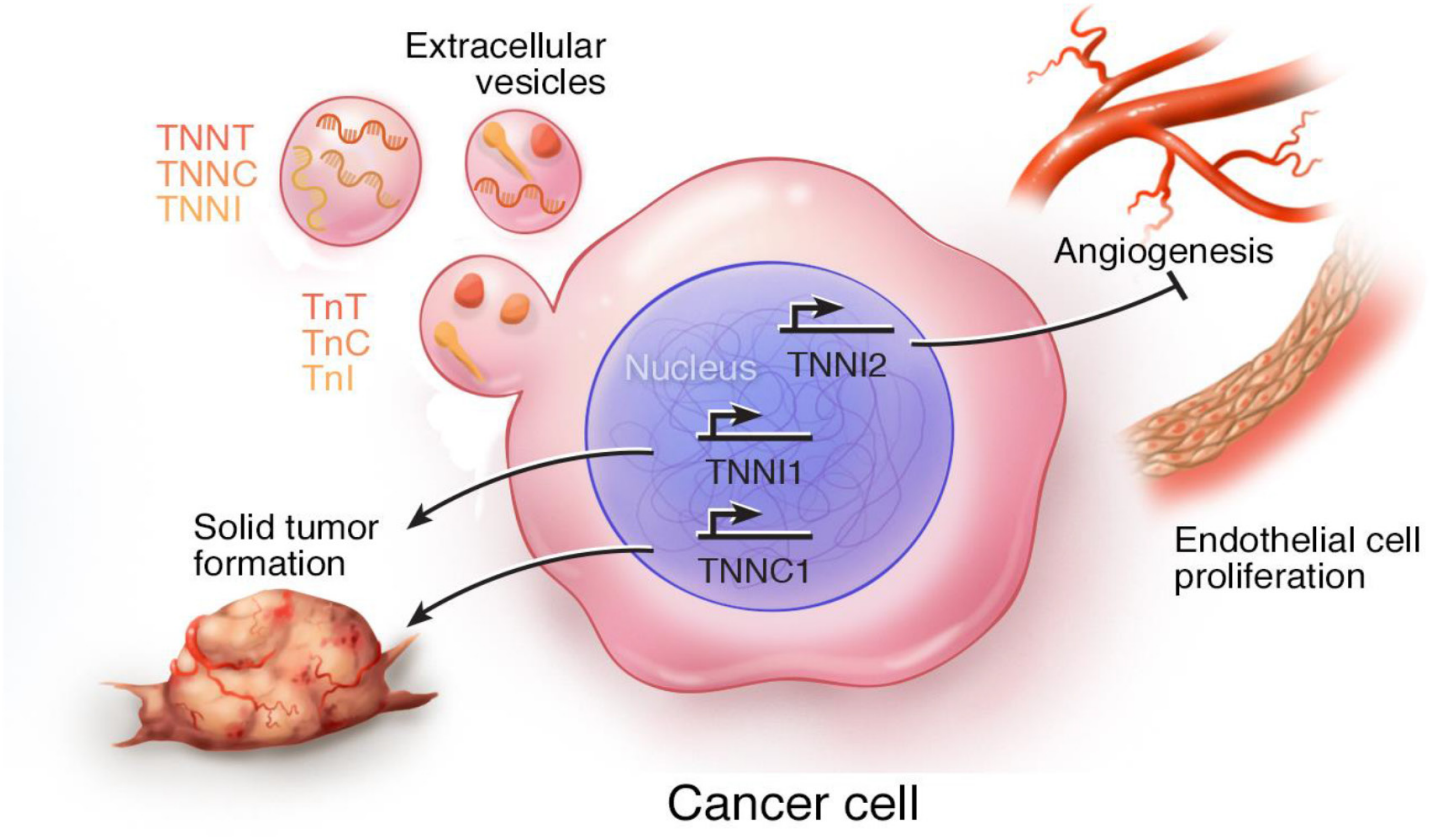
Schematic summary of the cellular processes associated with expression of troponin subunits in cancer cells Troponin subunit mRNAs and proteins have been identified in extracellular vesicles [33, 34, 106]. Fast skeletal TnI (*TNNI2*) inhibits (T-bar) endothelial cell proliferation and angiogenesis [27, 84–86]. Overexpression of *TNNC1* and *TNNI1* promotes (arrow bar) solid tumor growth and metastasis [23, 72, 73].

Cancer is a devastating illness worldwide and remains the second leading cause of death in the United States, after cardiovascular disease [107]. Based on the synthesis of current findings, expression of troponin subunits seems to be associated with aggressive, often metastatic, forms of cancer. It appears that the effects of troponin are multifaceted and isoform-dependent. For example, ssTnI and cTnC enhance malignant cell growth [23, 25], while fsTnI attenuates tumor growth [27, 85, 86]. The cellular processes associated with troponin subunit expression in cancer cells are summarized schematically in Figure 4. It has been previously shown that expression of *TNNI2* inhibits endothelial cell proliferation and angiogenesis (indicated by the T-bar, Figure 4), whereas expression of *TNNC1* and *TNNI1* promote tumor growth and metastasis (indicated by arrows, Figure 4). Alternatively, the functional relevance of troponin mRNAs and protein subunits in extracellular vesicles is currently unknown; however, given their presence in EVs secreted from cancer cells, we favor a theory that implicates their involvement in tumor growth and metastasis.

Results obtained from *in vivo* experiments on small rodent tumor models provide compelling support for the prospect of troponin-based, targeted therapies to combat tumorigenesis and metastasis in certain cancers. It should be recognized, however, that targeting troponin might present substantial challenges due to its vital regulatory role in the heart and skeletal muscles (e.g. diaphragm). Several chemotherapeutic agents are already known to induce cardiotoxicity [108, 109]. While blood plasma levels of troponin subunits cTnI and cTnT are currently used as diagnostic markers for myocardial infarction [110–112], troponin may also have potential diagnostic and prognostic utility in oncology as a genetic biomarker for a subset of cancers [113]. Despite the modest progress made in efforts to understand troponin’s role(s) in cancer, many questions remain unanswered. For instance, it is unclear whether TnC, TnI and TnT function within the ternary complex (as in the muscle sarcomere) and/or with non-troponin proteins in cancer cells, or function independently as individual subunits/proteolytic fragments. Also, the intracellular versus extracellular contributions to the effects observed in cancer cells need to be clarified. Furthermore, detailed mechanistic studies on the isoform differences and spatial-temporal expression patterns of troponin subunits (both gene and protein) are needed to determine the functional significance of this regulatory protein in cancer cells. Since troponin is a component of the molecular cargo in cancer cell-derived EVs, it begs the question: is troponin another “seed in the soil” of tumor microenvironments?

## TROPONIN: MULTITASKING IN THE MUSCLE CELL

In addition to its well-established function in the myofilament, growing evidence indicates troponin subunits have multiple functions beyond regulating muscle contraction. Thus far, these functions have been implicated in novel mechanisms of cardiomyopathy pathogenesis and skeletal muscle aging. Below we highlight the recent findings of troponin multitasking in compartments beyond the contractile apparatus of striated muscle cells.

### Nuclear localization of troponin

Several lines of evidence have challenged the longstanding view that troponin is solely a myofilament protein, restricted to thin filaments of sarcomeres that are located in a striated muscle cell’s cytoplasmic compartment. Since the discovery of chromosome-localized troponin and Tm in non-muscle cells of *Drosophila* [58], several studies have demonstrated the presence of troponin, along with other myofilament proteins, in the nuclear compartment of cardiac and skeletal muscle cells (see [38] for detailed review). Briefly, the first piece of evidence that demonstrated nuclear-associated troponin emanated from a seminal study on cardiomyocyte renewal capacity [36]. Although it was initially unclear whether the troponin subunits were on the surface or within the nucleus, we have previously shown by confocal microscopy (z-stack optical sectioning) that a pool of troponin subunits are localized within the nuclei of neonatal rat ventricular cardiomyocytes [35]. Moreover, a proteomics study on enriched organelles indicated that troponin subunits are associated with distinct sub-nuclear compartments in rodent cardiomyocyte nuclei [114].

The possibility of cytoplasmic spillover into the nucleus is not entirely unfounded. Sample type (e.g. diseased *vs.* healthy), treatments, and preparation may result in structurally compromised organelles. While it is reasonable to speculate that these observations might be an artifact owing to cytoplasmic contamination, it is unlikely for reasons described previously [38, 115]. Furthermore, the existence of nuclear-localized troponin is supported by the observation that TnT and TnI isoforms both contain multiple nuclear localization sequences [36, 38, 116]. Perhaps the most compelling evidence supporting the presence of nuclear-localized troponin in striated muscle cells arises from recent insights implicating functional roles in skeletal muscle aging and cardiomyopathy pathogenesis.

### TnT as a transcription factor and its roles in aging skeletal muscle

The classical role of TnT is to anchor the troponin complex to the thin filament and participate in excitation-contraction coupling (ECC) in striated muscle cells [5]. However, in skeletal myocytes, it appears that TnT regulates multiple cellular processes beyond contraction. Several reports have implicated nuclear-localized fsTnT (TnT3) in aging-associated sarcopenia—an adverse condition marked by progressive decline in skeletal muscle strength during normal aging. In an elegant study using fluorescent TnT3 constructs in various cell lines (NIH3T3 fibroblasts, C2C12 myoblasts), it was demonstrated that full length (and fragments of) TnT3 localize to the nucleus, and are closely associated with RNA polymerase activity and nucleolar regions [39].

The association between TnT and transcription-related processes should not be surprising, since other cytoskeletal proteins (i.e. actin and myosin) have been shown to regulate transcriptional processes [50, 52, 55]. This study also showed that overexpression of TnT3 fragments resulted in morphological alterations of nuclear structures and augmented apoptosis in an age-dependent manner. This observation is consistent with a previous report of TnT fragments inducing apoptosis [117]. Overall, these studies provide clear evidence of a link between nuclear TnT3 and sarcopenia-related processes. In an effort to explore the underlying molecular mechanisms of TnT3 nuclear localization and apoptosis in muscle cells, the same group performed transfection experiments with modified TnT constructs [41]. It was demonstrated that a nuclear/nucleolar localization signal present on the carboxy-terminus of TnT3 fragment and leucine zipper DNA-binding domain are important for mediating nuclear signaling and apoptosis in muscle cells. Based on these observations, it was proposed these processes likely contribute to the muscle weakness in age-related sacropenia [41]. Not only do these studies suggest a novel function for TnT3 (i.e. transcription factor) in muscle cells, but they also provide a potential mechanistic basis for the previously unexplained muscle weakness observed in age-related sarcopenia.

ECC in muscle cells is regulated by a variety of proteins, including the voltage-gated calcium channel subunit (Ca_v_β_1a_) [118]. In addition to the classical role of Ca_v_β_1a_ as part of the dihydropyridine receptor that is the voltage sensor in the transverse-tubule membrane for Ca^2+^ release from the sarcoplasmic reticulum, it has also been shown to localize to the nucleus of muscle progenitor cells and regulate gene expression [119]. In an effort to understand the molecular mechanisms governing the translocation, the same group subsequently demonstrated that TnT3 facilitates the nuclear recruitment of Ca_v_β_1a_ [40]. These results are consistent with a previous report demonstrating nuclear-localized troponin early in muscle cell differentiation [35]. The functions of full length TnT3 and its fragments extend beyond ECC and shuttling Ca_v_β_1a_ to the nuclear compartment of skeletal muscle cells. As previously described, the presence of a DNA-binding domain on TnT3 and its nuclear localization suggested this subunit might be a transcription factor [41]. The same group went on to show that TnT3 directly regulates Ca^2+^ α1 subunit (Cav1.1) gene expression, another component of the dihydropyridine receptor in the transverse-tubule membrane which is involved in ECC [120]. Specifically, it was observed that intra-nuclear accumulation of C-terminal fragments from TnT3 (which contains the DNA-binding domain) resulted in decreased expression of Cav1.1 and impaired ECC with aging in mice. In this study, the DNA-binding activity of TnT3 was mapped to the promoter region of the Cav1.1 gene.

Reduced expression of Cav1.1 leads to uncoupling of excitation and contraction, and is associated with age-related functional decline in skeletal muscle [120]. The authors went on to show that ECC dysfunction could be rescued in sedentary, old mice by administration of an inhibitor of TnT3 fragmentation—presumably by a mechanism that inhibits fragmented TnT3-dependent downregulation of Cav1.1 expression. Recent findings revealed that cTnT is enriched at the neuromuscular junction in fast-twitch muscle fibers of older mice [121]. Based on several observations in this study, the authors proposed that cTnT contributes to skeletal muscle dysfunction and denervation through mechanisms that involved PKA signaling in aging mice. Taken together, these studies illustrate non-classical roles for TnT and establish evidence supporting its role as a transcription factor.

### Functional insights into troponin’s nuclear role in cardiomyocytes

Nuclear localization of troponin is not exclusive to skeletal muscle cells. Although most functional studies on intranuclear troponin have focused on skeletal muscle cells, evidence supporting a role in the cardiomyocyte nucleus is beginning to emerge—particularly in the setting of cardiomyopathy pathogenesis. Mutations in troponin are known to cause various forms of inherited cardiomyopathy [15, 122]. Though it is known that functional perturbations in the contractile apparatus and myofilament Ca^2+^ sensitivity contribute to the pathogenesis of inherited cardiomyopathies [123–136], additional mechanisms involving nuclear troponin signaling have emerged. In an effort to understand the molecular basis of epigenetic modifications observed in a human induced pluripotent stem cell (iPSC) model of inherited dilated cardiomyopathy (DCM), one group reported nuclear accumulation of cTnT in DCM cells compared to control cells [37]. Immunoprecipitation and mass spectrometry experiments revealed that TnT might interact–either directly or indirectly—with key histone demethylases, KDM1A and KDM5A, and affect epigenetic modifications [37]. Overall, this study implicated a functional role for nuclear cTnT and unveiled a novel mechanism by which a mutation in the cTnT gene contributes to DCM pathogenesis.

In a study on arsenic-induced cardiac hypertrophy in rats, bioinformatics analyses implicated cTnI in signaling pathways associated with cardiac remodeling [137]. Specifically, the pathway analysis revealed potential interactions between cTnI and known transcription factors that mediate hypertrophy [137]. These findings suggest the possibility of a signal-dependent role for nuclear cTnI in cardiac hypertrophy. In another report, cTnI was identified in the nuclei, but not the cytoplasm of mesenchymal stem cells (MSCs) treated with the histone deacetylase inhibitor, apicidin [138]. Apicidin treatment was associated with commitment of MSCs to the cardiac lineage—which, when combined with untreated MSCs, lead to improved cardiac function in an animal model of myocardial infarction. In this study, a mechanism involving YAP/miR-130a was invoked [138]. Based on these findings and a previous report [35], it is enticing to speculate that nuclear cTnI, along with other troponin subunits, might be involved in the processes related to cardiomyocyte differentiation.

### Troponin I: implications for immune signaling

An additional line of evidence that hints at a role for troponin beyond muscle contraction derives from a series of studies on immune responses in humans and rodents, post-myocardial infarction (MI). The release of troponins into the blood post-MI is associated with an immune response involving the production of autoantibodies [139, 140]. It was previously shown that cTnI specifically induces autoimmune inflammation of the myocardium [141]. To further understand how cTnI provokes autoimmunity, wild-type mice were immunized with specific cTnI peptides and cardiac function was assessed, along with various indicators of immune response [140]. Interestingly, two cTnI peptides, but not other regions of cTnI tested, induced significant myocardial inflammation and fibrosis in the mice, which subsequently progressed to heart failure and death [140]. Mice immunized with the two peptides independently mounted a robust autoimmune response, marked by a significant increase in expression of chemokines [140]. From a structural perspective, the antigenic cTnI peptides correspond to TnT-binding regions in the troponin complex [1]. Of importance, peptides corresponding to the slow skeletal isoform of TnI failed to elicit a significant immune response, suggesting isoform specificity of cTnI in autoimmune signaling. A detailed mechanistic basis for these observations is lacking, but it is clear that cTnI seems to be involved in immune system signaling. It would be worthwhile to determine if a fraction of full length TnI and/or fragments elicit an immune response via signaling in the nucleus, similar to TnT3 in aging skeletal muscle.

### Outlook on troponin in muscle cells

It is well known that the troponin complex regulates striated muscle cell contraction in the heart and skeletal tissue; however, the discovery of troponin subunits in the nuclear compartment of muscle cells has led to intriguing findings of additional functions for troponin. While it was initially assumed that troponin in the nucleus of muscle cells was merely an artifact due to cytoplasmic contamination, the emerging functional roles suggest otherwise. Nevertheless, it is clear that troponin is multitasking in striated muscle cells. In skeletal muscle cells, fsTnT/fragments have been shown to regulate apoptosis [41, 117] and function as a transcription factor to regulate genes involved in ECC [40]. These findings not only demonstrate the first piece of evidence supporting a function for a nuclear-localized troponin subunit in muscle cells, but they also revealed remarkable relevance to the consequences of aging such as sarcopenia. Based on these key findings, it has been suggested that modulating calpain activity and reducing TnT3 fragmentation are potential therapeutic strategies for ameliorating age-related deterioration in muscle strength [142]. In patient-derived iPSC cardiomyocytes bearing a DCM-associated mutation in *TNNT2*, it was shown that cTnT accumulates in the nucleus and may regulate epigenetic modifications that exacerbate the pathogenesis of cardiomyopathy [37]. Despite the difference in TnT isoforms (cardiac *vs.* skeletal), results from the skeletal muscle cells and cardiomyocytes both suggest TnT’s involvement in transcription and nucleolar processes.

Another study uncovered a novel role for cTnI, where it was implicated in arsenic-induced hypertrophic signaling and interactions with several important transcription factors [137]. Furthermore, nuclear cTnI was found in the nucleus of MSCs during commitment to the cardiac lineage [138]. Finally, cTnI was implicated in autoimmune signaling and peptides of cTnI were shown to induce myocardial inflammation and fibrosis in mice [140]. Based on the aforementioned findings, we speculate that nuclear-localized troponin is involved in these diverse cellular processes. Although our current understanding of troponin beyond muscle contraction is predominantly in the context of disease and aging, it does not seem premature to speculate on whether troponin is involved in early embryonic development via Ca^2+^-dependent processes in the nuclear compartment. Indeed, Ca^2+^ signaling has been implicated in cardiomyocyte transcriptional regulation [143]. The striking observation of nuclear troponin appearing early in bone marrow-derived stem cells stimulated to differentiate certainly provides merit to this conjecture [35]. Furthermore, in a large-scale screen examining genes controlling murine embryogenesis, genes encoding multiple troponin subunits were identified [144].

The functions of skeletal muscle troponin isoforms in cancer cells, reviewed above, have implications for muscle physiology. If fsTnI inhibits angiogenesis, then perhaps this could at least be partially responsible for lower capillary density observed in fast skeletal muscle fibers relative to slow skeletal fibers that express the ssTnI isoform [145]. With skeletal muscles being chock-full of TnI protein, how do they maintain their normal physiology as myocytes, and not succumb to the problems of pathophysiology? The coordinated expression of troponin subunits (in terms of thin filament protein stoichiometry) may represent one straightforward, plausible explanation, although there may be other mechanisms specific to myocytes. Ongoing work in our group is focused on understanding the functional role of nuclear-localized troponin in the heart during health and disease. Overall, it is becoming clear that troponin is more than just a regulator of contraction in striated muscle cells.

## BIOINFORMATICS ANALYSIS PROVIDES MECHANISTIC INSIGHT INTO TROPONIN’S DIVERSE CELLULAR FUNCTIONS

Most of the studies described above have advanced our understanding of the functional roles for troponin beyond muscle contraction; however, the underlying mechanistic basis for most of these proposed functions is poorly understood. Therefore, we utilized several bioinformatics approaches to seek deeper mechanistic insight and stimulate further scientific inquiry. Findings from pathway, protein-protein, and protein-DNA analyses support a role for troponin functioning in both the cytoplasmic and nuclear compartments, with implications for processes related to cell differentiation, growth, and proliferation.

### Pathway analysis

**Figure 5:**
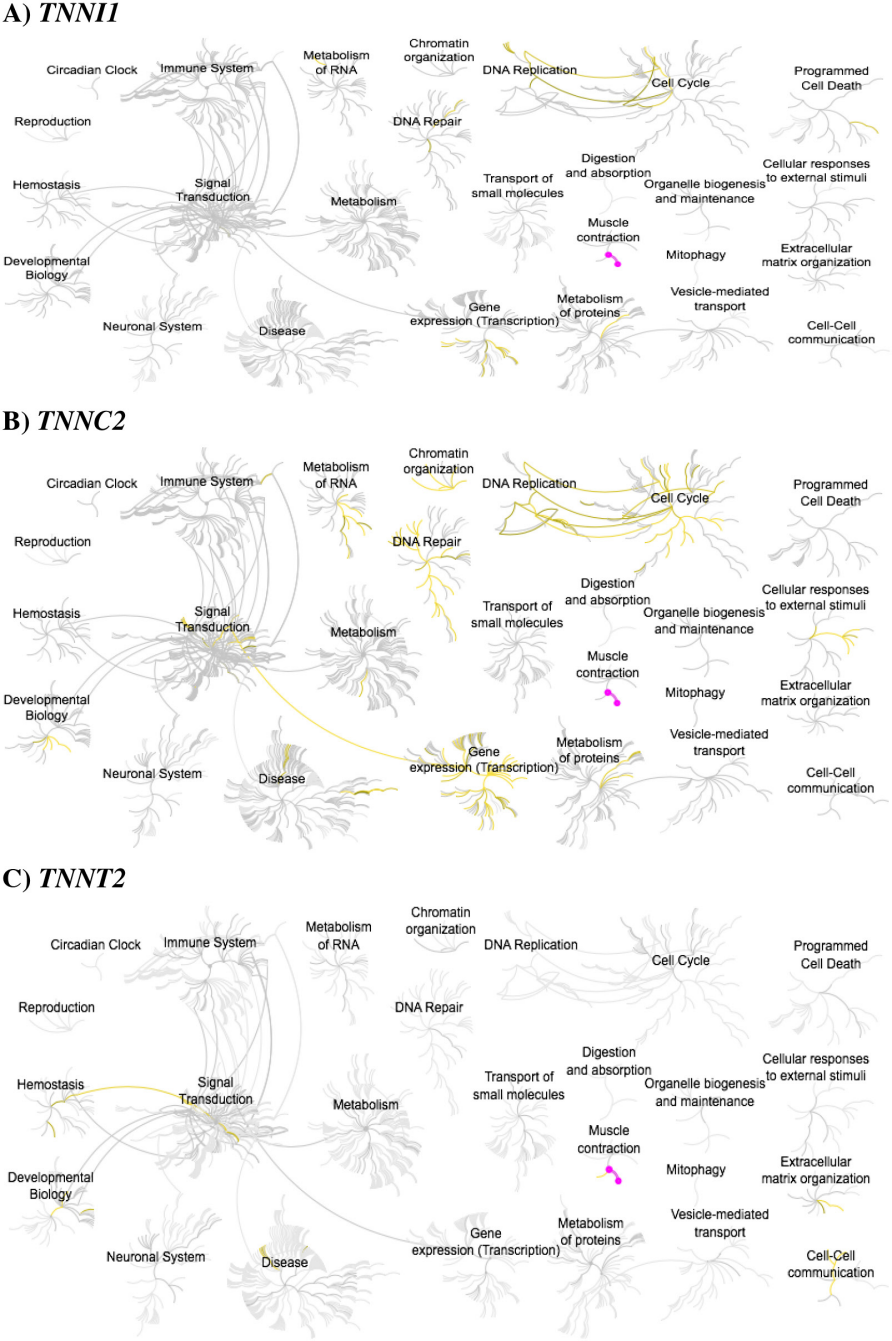
Reactome pathway analysis for three troponin genes **(A)** *TNNI1* **(B)** *TNNC2* and **(C)** *TNNT2*. Gold and magenta lines indicate flagged pathways based on the analysis. Images from the Reactome database (http://reactome.org) were accessed through the Human Protein Atlas database (http://www.proteinatlas.org) in August 2017.

The identification of troponin subunits in the nuclear compartment is a novel finding. Therefore, our current understanding of its potential role(s) in the nucleus remains poorly understood. To provide mechanistic insight into the potential functions of nuclear-localized troponin subunits, we utilized Reactome—an open source, peer reviewed pathway analysis database [146, 147]. For each of the troponin subunits examined, the outputs all contain a strong association with the pathway of “muscle contraction,” as expected, thus serving as a positive control for the analysis. Pathway analysis of *TNNI1* reveals a direct link between the central nodes of “cell cycle” and “DNA replication,” as well as associations with “DNA repair,” “programmed cell death,” and “gene expression (transcription)” nodes (Figure 5A). If ssTnI is involved in processes such as DNA replication and repair, then accumulating somatic mutations in *TNNI1* may contribute to cancer development by perturbing genomic stability. *TNNC2* pathway analysis shares the trend observed for *TNNI1*, but with the addition of “chromatin organization,” “developmental biology,” “signal transduction,” and “metabolism of RNA” (Figure 5B). Interestingly, subpathway analysis emanating from the central node (gold lines) of “developmental biology” reveals implications in HOX gene activation during differentiation and transcriptional regulation of pluripotent stem cells.

*TNNT2* is implicated in “cell-cell communication,” “extracellular matrix organization,” and a linked pathway between the central nodes of “signal transduction” and hemostasis” via integrin signaling (Figure 5C). Although the database provides evidence of *TNNT2* in the nucleus, pathway analysis was only available for its association with focal adhesion sites. Nevertheless, both compartments are important to consider in the context of cell division and it is likely that *TNNT2* is associated with pathways in the nucleus. Overall, these pathways are connected to cell growth, differentiation, and proliferation—with a majority of processes enriched in the nuclear-compartment. Based on these *in silico* findings, we speculate that troponin subunits may contribute to tumorigenesis and cardiomyopathy pathogenesis by functioning in processes related to the cell cycle, transcription, and DNA replication.

### Troponin’s putative interacting partners: from proteins to nucleic acids

In addition to the pathway analysis, examination of troponin’s potential interacting partners provides insight that is consistent with functions in the nuclear compartment of the cell. Utilizing the BioGrid (3.4) protein-protein interaction database accessed through UniProt (http://www.uniprot.org), we have tabulated the putative interacting partners based on data from two-hybrid assays for individual human troponin subunits (Table 1). For brevity, we excluded known interacting partners found in the contractile apparatus (i.e. Tm, actin, etc.). It is important to note that these interactions are considered putative due to the inherent limitations of high throughput assays and dearth of experimental validation. In spite of this, the putative partners are remarkably consistent with results from the pathway analysis and previously reported functional studies. For example, cTnC putatively interacts with cyclin-dependent kinase 1 (CDK1), indicating its potential involvement in cell cycle regulation (Table 1). This is not only consistent with the pathway analysis implicating cTnC in the cell cycle, but is also consistent with its growth-promoting effects *in vivo* [70, 71]. Moreover, this isoform of TnC ostensibly interacts with the G subunit of RNA polymerase II (POLR2G)—suggesting a potential role in mRNA synthesis (Table 1). Interestingly, cTnC also putatively interacts with non-muscle myosin isoforms, which were implicated in transcription [50, 52, 55].

**Table 1:** Putative protein-protein interactions for human troponin subunits

Troponin Subunit	Putative Interacting Partners
Slow-skeletal/cardiac Troponin C	POLR2G, CDK1, TRIM63, MYO5B, MIDN, XRCC6BP1, FBXO30, MYO1B, MYO3A, MYO3B, MYO5A, RBM15B, UBB, UBE2C, TCP10L, CNN1, IQCD
Fast skeletal troponin C	NOSIP, SSFA2, MAP7D3, MTPAP, NEDD8-MDP1, SMARCD3, ATF7IP, ZBTB21, SEPHS2, POGZ, HIRIP3, ARNT2, RPS6KA4, PAX6, C7ORF33, MECOM, GDF5, MAML1, CLSPN, PML, CHAMP1, PIK3R3, KCNAB2, SNRPC, UBB, ACTA2, CALML3, SPECC1L, NRIP1, IQGAP1, ITPR2, ITPR3, KIF20A, ZNF74, DCAFF6, MAGED2, ECI2, TTC6
Slow-skeletal Troponin T	PRKG1, TMP1, ARMC8, PSMC5, LARP1, FAF1, DDX5, CHD3, FYN, C2ORF44, TPM3, SEC31A, HAP1, VIM, NFE2L2, LDOC1, TBPL1, MORF4L1, TMEM98, TFIP11, CCDC136, KRT40, TLK1, TLK2, CDK5RAP2, MAEA, RMND5A, GID4, TJP2, MARS, OSBP2, TRA2A, NACAD, PLEKHF1, PPFIA1, ZMYND19, NINL, FXR2, TRIM63, IMMT, SH3GL3, ZNF768, KAT5, PI4KA, EEF1G, HSP90AB1, HMGXB4, BLOC1S2, SNW1, ZC3H15, HMP19, SERPINA4, UBE2D1, ZNF250, ZKSCAN5, NAGK, OSM, CCDC85B, RNF41
Cardiac Troponin T	APP, MORF4L2, NOTCH3
Fast-skeletal Troponin T	PGPEP1, SNUPN, GID4, MAEA, ARMC8, TXLNG, YTHDC2, NLGN3, HAP1, NUDT3, TSG101, TRIM63, ZMYND19,
Slow-skeletal Troponin I	TRIM63, CCDC85B, PDK2L1, TRIM55, MDFI, PNMA1, MYH3, MYH6, MYH7, ACTN2, TPM1, TPM2,
Fast-Skeletal Troponin I	TRIM63, RORB, HNF4G, ESRRA, UPF2, TRIM55, NUCB2, RFTN2, KLHL21, TLE4, TLE1, SIRT1, MECOM, PGPEP1
Cardiac Troponin I	PKD2L1, PKD2, RCAN3, TNNI3K, TRIM63, LYST, TRIM55, SOX4, PSMC5, HSPB2, AKAP1, IFNA4

Summary of potential interacting partners for each troponin subunit was obtained through the BioGRID (3.4) database accessed via UniProt. For brevity, known sarcomeric protein interactors for each troponin subunit were omitted (e.g. Tm and actin).

The fsTnC isoform also putatively interacts with proteins associated with the nucleus. For instance, potential interacting partners include activating transcription factor 7 interacting protein (ATF7IP), small nuclear ribonucleoprotein polypeptide C (SNRPC), promyelocytic leukemia protein (PML), and MDS1-EVI1 complex locus protein (MECOM) (Table 1). Consistent with the Reactome analysis, fsTnC putatively interacts with chromatin organization proteins, such as SMARCD3 and HIRIP3 (Table 1). cTnT putatively interacts with NOTCH3, a protein key to neuronal development (Table 1). On the other hand, fsTnT putatively interacts with a cytosolic protein implicated in tumorigenesis, TSG101 (Table 1). TnI isoforms potentially interact with nuclear proteins, such as TNNI3K, SOX4, and transducin-like enhancers, TLE4/TLE1 (Table 1). Many of troponin’s putative interacting partners are known transcription factors, which suggests a role in transcriptional activity. Based on these observations, we performed a DNA-binding prediction for each troponin subunit using the FASTA files (Table 2). Isoforms of TnI and TnT, but not TnC, are strongly predicted to bind DNA (Table 2). This is not surprising since TnT has been shown to function as a transcription factor [120] and shares core structural similarity with TnI isoforms [6]. Furthermore, the structures of TnT and TnI, along with their enrichment of basic residues, favor the notion of these subunits interacting with nucleic acids (Figure 1). Therefore, one potential mechanism by which troponin subunits may orchestrate diverse cellular processes (e.g. cell growth and apoptosis) is by functioning as a transcription factor. Furthermore, troponin interactions with RNA seem equally conceivable.

**Table 2:** DNA binding prediction for troponin subunits

Troponin Subunit	Uniprot ID	Score	Probability of DNA binding	Prediction
Slow skeletal/cardiac troponin I	P19237	6.452	0.9984	DNA-binding
Fast skeletal troponin I	P48788	5.424	0.9956	DNA-binding
Cardiac troponin I	P19429	6.416	0.9984	DNA-binding
Slow skeletal troponin T	P13805	6.154	0.9979	DNA-binding
Cardiac troponin T	P45379	6.751	0.9988	DNA-binding
Fast skeletal troponin T	P45378	5.904	0.9973	DNA-binding
Slow skeletal/cardiac troponin C	P63316	-3.978	0.0184	non-DNA-binding
Fast skeletal troponin C	P02585	-2.956	0.0494	non-DNA-binding

DNABIND prediction software [154] was used to predict DNA binding with a 5% false positive rate based on the primary sequences obtained from Uniprot.

## CONCLUDING REMARKS: IS TROPONIN A MOONLIGHTING PROTEIN?

Most proteins in a given cell have one primary function, but there are several examples of proteins that have multiple, often unrelated, functions—known as moonlighting proteins [148, 149]. It is time to recognize that troponin has several functions beyond muscle contraction, with profound implications in cancer, cardiomyopathy pathogenesis, and age-related processes. Furthermore, it is clear that troponin plays a key role in age-related processes. Despite the recent advances uncovering the non-canonical roles of troponin, many challenges and unresolved questions remain. For instance, is troponin functioning as a complex or individual subunits in cancer cells, and in cardiomyocyte nuclei? Given that TnI is a binding partner of TnC in muscle cells [150] and is inherently unstable (in terms of proteolytic susceptibility and solubility) as an individual subunit [151, 152], it is reasonable to surmise that TnI may also function in complex with TnC and TnT outside of the myofilament apparatus. Does troponin function as a transcription factor in cardiomyocytes and cancer cells? Given that troponin subunits contain DNA-binding domains and nuclear localization signals, its role as a transcription factor seems likely beyond what has already been established. Furthermore, troponin contains several regions of intrinsic disorder [1, 2, 151, 153] and a high proportion of basic residues, which may contribute to its molecular promiscuity and multifaceted functions in the cell. Future studies should focus on distinguishing intracellular (specifically subcellular) versus extracellular effects of troponin, as well as isoform differences.

What molecular mechanisms govern the ability of fsTnT to inhibit angiogenesis? This question has far-reaching implications for harnessing fsTnT as an anti-tumor therapeutic. Why is troponin packaged into extracellular vesicles secreted by cancer cells? Perhaps this represents a mechanism by which malignant tumors acquire metastatic potential. Does *TNNC1* function as a proto-oncogene? Accumulating evidence suggests this possibility, but definitively classifying it as such will require additional comprehensive studies. Nonetheless, it is clear that troponin genes are often overexpressed in certain cancers—a fundamental observation that provides a novel avenue for exploring the molecular mechanisms of tumorigenesis. The multifunctional properties of troponin indicate that it has been endowed with exquisite functional diversity. Perhaps additional moonlighting functions will emerge, so long as they are not eclipsed by the parochial view that troponin’s sole duty is to regulate striated muscle contraction.

## SUPPLEMENTARY MATERIALS TABLES





## Abbreviations

cTnC: Slow Skeletal/Cardiac Troponin C
fsTnC: Fast Skeletal TnC
*TNNC1*: Slow Skeletal/Cardiac Troponin C gene
*TNNC2*: Fast Skeletal Troponin C gene
fsTnT/TnT3: Fast Skeletal Troponin T
ssTnT: Slow Skeletal Troponin T
cTnT: Cardiac Troponin T
*TNNT1*: Slow Skeletal Troponin T gene
*TNNT2*: Cardiac Troponin T gene
*TNNT3*: Fast Skeletal Troponin T gene
cTnI: Cardiac Troponin I
ssTnI: Slow Skeletal Troponin I
fsTnI: Fast Skeletal Troponin I
*TNNI1*: Slow Skeletal Troponin I gene
*TNNI2*: Fast Skeletal Troponin I gene
*TNNI3*: Cardiac Troponin I gene
*TNNI3K*: Cardiac Troponin I-Interacting Kinase gene
Tm: Tropomyosin
EVs: Extracellular Vesicles
ECC: Excitation-Contraction Coupling
DCM: Dilated Cardiomyopathy
COSMIC: Catalogue of Somatic Mutations in Cancer database

## References

[1] Takeda S, Yamashita A, Maeda K, Maeda Y (2003). Structure of the core domain of human cardiac troponin in the Ca2+-saturated form. Nature.

[2] Vinogradova MV, Stone DB, Malanina GG, Karatzaferi C, Cooke R, Mendelson RA, Fletterick RJ (2005). Ca2+-regulated structural changes in troponin. Proc Natl Acad Sci U S A.

[3] Gahlmann R, Kedes L (1990). Cloning, structural analysis, and expression of the human fast twitch skeletal muscle troponin C gene. J Biol Chem.

[4] Schreier T, Kedes L, Gahlmann R (1990). Cloning, structural analysis, and expression of the human slow twitch skeletal muscle/cardiac troponin C gene. J Biol Chem.

[5] Wei B, Jin JP (2016). TNNT1, TNNT2, and TNNT3: Isoform genes, regulation, and structure-function relationships. Gene.

[6] Sheng JJ, Jin JP (2016). TNNI1, TNNI2 and TNNI3: Evolution, regulation, and protein structure-function relationships. Gene.

[7] Katrukha IA (2013). Human cardiac troponin complex. Structure and functions. Biochemistry (Mosc).

[8] Farah CS, Reinach FC (1995). The troponin complex and regulation of muscle contraction. Faseb J.

[9] Gomes AV, Potter JD, Szczesna-Cordary D (2002). The role of troponins in muscle contraction. IUBMB Life.

[10] Tobacman LS (1996). Thin filament-mediated regulation of cardiac contraction. Annu Rev Physiol.

[11] Ebashi S, Kodama A (1965). A new protein factor promoting aggregation of tropomyosin. J Biochem.

[12] Ebashi S, Kodama A (1966). Interaction of troponin with F-actin in the presence of tropomyosin. J Biochem.

[13] Chang AN, Parvatiyar MS, Potter JD (2008). Troponin and cardiomyopathy. Biochem Biophys Res Commun.

[14] Gomes AV, Potter JD (2004). Molecular and cellular aspects of troponin cardiomyopathies. Ann N Y Acad Sci.

[15] Willott RH, Gomes AV, Chang AN, Parvatiyar MS, Pinto JR, Potter JD (2010). Mutations in Troponin that cause HCM, DCM AND RCM: what can we learn about thin filament function?. J Mol Cell Cardiol.

[16] Lu QW, Wu XY, Morimoto S (2013). Inherited cardiomyopathies caused by troponin mutations. J Geriatr Cardiol.

[17] Parvatiyar MS, Pinto JR, Dweck D, Potter JD (2010). Cardiac troponin mutations and restrictive cardiomyopathy. J Biomed Biotechnol.

[18] Kinoshita S, Adachi W, Sotozono C, Nishida K, Yokoi N, Quantock AJ, Okubo K (2001). Characteristics of the human ocular surface epithelium. Prog Retin Eye Res.

[19] Fine R, Lehman W, Head J, Blitz A (1975). Troponin C in brain. Nature.

[20] Berezowsky C, Bag J (1992). Slow troponin C is present in both muscle and nonmuscle cells. Biochem Cell Biol.

[21] Lowe XR, Lu X, Marchetti F, Wyrobek AJ (2007). The expression of Troponin T1 gene is induced by ketamine in adult mouse brain. Brain Res.

[22] Lyckman AW, Horng S, Leamey CA, Tropea D, Watakabe A, Van Wart A, McCurry C, Yamamori T, Sur M (2008). Gene expression patterns in visual cortex during the critical period: synaptic stabilization and reversal by visual deprivation. Proc Natl Acad Sci U S A.

[23] Leung CS, Yeung TL, Yip KP, Pradeep S, Balasubramanian L, Liu J, Wong KK, Mangala LS, Armaiz-Pena GN, Lopez-Berestein G, Sood AK, Birrer MJ, Mok SC (2014). Calcium-dependent FAK/CREB/TNNC1 signalling mediates the effect of stromal MFAP5 on ovarian cancer metastatic potential. Nat Commun.

[24] Chen C, Liu JB, Bian ZP, Xu JD, Wu HF, Gu CR, Shi Y, Zhang JN, Chen XJ, Yang D (2014). Cardiac troponin I is abnormally expressed in non-small cell lung cancer tissues and human cancer cells. Int J Clin Exp Pathol.

[25] Casas-Tintó S, Maraver A, Serrano M, Ferrús A (2016). Troponin-I enhances and is required for oncogenic overgrowth. Oncotarget.

[26] Zhu X, Wang F, Zhao Y, Yang P, Chen J, Sun H, Liu L, Li W, Pan L, Guo Y, Kou Z, Zhang Y, Zhou C (2014). A gain-of-function mutation in Tnni2 impeded bone development through increasing Hif3a expression in DA2B mice. PLoS Genet.

[27] Kern BE, Balcom JH, Antoniu BA, Warshaw AL, Fernández-del Castillo C (2003). Troponin I peptide (Glu94-Leu123), a cartilage-derived angiogenesis inhibitor: *in vitro* and *in vivo* effects on human endothelial cells and on pancreatic cancer. J Gastrointest Surg.

[28] Gabbiani G, Trenchev P, Holborow EJ (1975). Increase of contractile proteins in human cancer cells. Lancet.

[29] Gabbiani G, Csank-Brassert J, Schneeberger JC, Kapanci Y, Trenchev P, Holborow EJ (1976). Contractile proteins in human cancer cells. Immunofluorescent and electron microscopic study. Am J Pathol.

[30] Li Y, Chen B, Chen J, Lou G, Chen S, Zhou D (2008). Fast skeletal muscle troponin I is a co-activator of estrogen receptor-related receptor alpha. Biochem Biophys Res Commun.

[31] Aboghe DH, Bolduc C, Yoshioka M, St-Amand J (2008). Effects of dihydrotestosterone on gene expression in mammary gland. J Steroid Biochem Mol Biol.

[32] Grewal JS, Bag J (1996). Slow troponin C gene expression in chicken heart and liver is regulated by similar enhancers. FEBS Lett.

[33] Hurwitz SN, Rider MA, Bundy JL, Liu X, Singh RK, Meckes DG (2016). Proteomic profiling of NCI-60 extracellular vesicles uncovers common protein cargo and cancer type-specific biomarkers. Oncotarget.

[34] Hong BS, Cho JH, Kim H, Choi EJ, Rho S, Kim J, Kim JH, Choi DS, Kim YK, Hwang D, Gho YS (2009). Colorectal cancer cell-derived microvesicles are enriched in cell cycle-related mRNAs that promote proliferation of endothelial cells. BMC Genomics.

[35] Asumda FZ, Chase PB (2012). Nuclear cardiac troponin and tropomyosin are expressed early in cardiac differentiation of rat mesenchymal stem cells. Differentiation.

[36] Bergmann O, Bhardwaj RD, Bernard S, Zdunek S, Barnabé-Heider F, Walsh S, Zupicich J, Alkass K, Buchholz BA, Druid H, Jovinge S, Frisén J (2009). Evidence for cardiomyocyte renewal in humans. Science.

[37] Wu H, Lee J, Vincent LG, Wang Q, Gu M, Lan F, Churko JM, Sallam KI, Matsa E, Sharma A, Gold JD, Engler AJ, Xiang YK (2015). Epigenetic Regulation of Phosphodiesterases 2A and 3A Underlies Compromised β-Adrenergic Signaling in an iPSC Model of Dilated Cardiomyopathy. Cell Stem Cell.

[38] Chase PB, Szczypinski MP, Soto EP (2013). Nuclear tropomyosin and troponin in striated muscle: new roles in a new locale?. J Muscle Res Cell Motil.

[39] Zhang T, Birbrair A, Wang ZM, Taylor J, Messi ML, Delbono O (2013). Troponin T nuclear localization and its role in aging skeletal muscle. Age (Dordr).

[40] Zhang T, Taylor J, Jiang Y, Pereyra AS, Messi ML, Wang ZM, Hereñú C, Delbono O (2015). Troponin T3 regulates nuclear localization of the calcium channel Cavβ1a subunit in skeletal muscle. Exp Cell Res.

[41] Zhang T, Birbrair A, Delbono O (2013). Nonmyofilament-associated troponin T3 nuclear and nucleolar localization sequence and leucine zipper domain mediate muscle cell apoptosis. Cytoskeleton (Hoboken).

[42] Bettinger BT, Gilbert DM, Amberg DC (2004). Actin up in the nucleus. Nat Rev Mol Cell Biol.

[43] Gonsior SM, Platz S, Buchmeier S, Scheer U, Jockusch BM, Hinssen H (1999). Conformational difference between nuclear and cytoplasmic actin as detected by a monoclonal antibody. J Cell Sci.

[44] Miyamoto K, Gurdon JB (2011). Nuclear actin and transcriptional activation. Commun Integr Biol.

[45] Dion V, Shimada K, Gasser SM (2010). Actin-related proteins in the nucleus: life beyond chromatin remodelers. Curr Opin Cell Biol.

[46] Hild G, Bugyi B, Nyitrai M (2010). Conformational dynamics of actin: effectors and implications for biological function. Cytoskeleton (Hoboken).

[47] Jockusch BM, Schoenenberger CA, Stetefeld J, Aebi U (2006). Tracking down the different forms of nuclear actin. Trends Cell Biol.

[48] Kandasamy MK, McKinney EC, Meagher RB (2010). Differential sublocalization of actin variants within the nucleus. Cytoskeleton (Hoboken).

[49] Pederson T, Aebi U (2002). Actin in the nucleus: what form and what for?. J Struct Biol.

[50] Almuzzaini B, Sarshad AA, Farrants AK, Percipalle P (2015). Nuclear myosin 1 contributes to a chromatin landscape compatible with RNA polymerase II transcription activation. BMC Biol.

[51] Philimonenko VV, Zhao J, Iben S, Dingová H, Kyselá K, Kahle M, Zentgraf H, Hofmann WA, de Lanerolle P, Hozák P, Grummt I (2004). Nuclear actin and myosin I are required for RNA polymerase I transcription. Nat Cell Biol.

[52] Ye J, Zhao J, Hoffmann-Rohrer U, Grummt I (2008). Nuclear myosin I acts in concert with polymeric actin to drive RNA polymerase I transcription. Genes Dev.

[53] Pestic-Dragovich L, Stojiljkovic L, Philimonenko AA, Nowak G, Ke Y, Settlage RE, Shabanowitz J, Hunt DF, Hozak P, de Lanerolle P (2000). A myosin I isoform in the nucleus. Science.

[54] Nowak G, Pestic-Dragovich L, Hozák P, Philimonenko A, Simerly C, Schatten G, de Lanerolle P (1997). Evidence for the presence of myosin I in the nucleus. J Biol Chem.

[55] Hofmann WA, Vargas GM, Ramchandran R, Stojiljkovic L, Goodrich JA, de Lanerolle P (2006). Nuclear myosin I is necessary for the formation of the first phosphodiester bond during transcription initiation by RNA polymerase II. J Cell Biochem.

[56] de Lanerolle P, Serebryannyy L (2011). Nuclear actin and myosins: life without filaments. Nat Cell Biol.

[57] Grummt I (2006). Actin and myosin as transcription factors. Curr Opin Genet Dev.

[58] Sahota VK, Grau BF, Mansilla A, Ferrús A (2009). Troponin I and Tropomyosin regulate chromosomal stability and cell polarity. J Cell Sci.

[59] Dingová H, Fukalová J, Maninová M, Philimonenko VV, Hozák P (2009). Ultrastructural localization of actin and actin-binding proteins in the nucleus. Histochem Cell Biol.

[60] Qi J, Chi L, Labeit S, Banes AJ (2008). Nuclear localization of the titin Z1Z2Zr domain and role in regulating cell proliferation. Am J Physiol Cell Physiol.

[61] McRobb LS, Lee VS, Simonian M, Zhao Z, Thomas SG, Wiedmann M, Raj JV, Grace M, Moutrie V, McKay MJ, Molloy MP, Stoodley MA (2017). Radiosurgery Alters the Endothelial Surface Proteome: Externalized Intracellular Molecules as Potential Vascular Targets in Irradiated Brain Arteriovenous Malformations. Radiat Res.

[62] Gao J, Aksoy BA, Dogrusoz U, Dresdner G, Gross B, Sumer SO, Sun Y, Jacobsen A, Sinha R, Larsson E, Cerami E, Sander C, Schultz N (2013). Integrative analysis of complex cancer genomics and clinical profiles using the cBioPortal. Sci Signal.

[63] Cerami E, Gao J, Dogrusoz U, Gross BE, Sumer SO, Aksoy BA, Jacobsen A, Byrne CJ, Heuer ML, Larsson E, Antipin Y, Reva B, Goldberg AP (2012). The cBio cancer genomics portal: an open platform for exploring multidimensional cancer genomics data. Cancer Discov.

[64] Supek F, Miñana B, Valcárcel J, Gabaldón T, Lehner B (2014). Synonymous mutations frequently act as driver mutations in human cancers. Cell.

[65] Uhlén M, Fagerberg L, Hallström BM, Lindskog C, Oksvold P, Mardinoglu A, Sivertsson Å, Kampf C, Sjöstedt E, Asplund A, Olsson I, Edlund K, Lundberg E (2015). Proteomics. Tissue-based map of the human proteome. Science.

[66] Hornbeck PV, Zhang B, Murray B, Kornhauser JM, Latham V, Skrzypek E (2015). PhosphoSitePlus, 2014: mutations, PTMs and recalibrations. Nucleic Acids Res.

[67] Mertins P, Yang F, Liu T, Mani DR, Petyuk VA, Gillette MA, Clauser KR, Qiao JW, Gritsenko MA, Moore RJ, Levine DA, Townsend R, Erdmann-Gilmore P (2014). Ischemia in tumors induces early and sustained phosphorylation changes in stress kinase pathways but does not affect global protein levels. Mol Cell Proteomics.

[68] Bowtell DD, Böhm S, Ahmed AA, Aspuria PJ, Bast RC, Beral V, Berek JS, Birrer MJ, Blagden S, Bookman MA, Brenton JD, Chiappinelli KB, Martins FC (2015). Rethinking ovarian cancer II: reducing mortality from high-grade serous ovarian cancer. Nat Rev Cancer.

[69] Ranzani M, Annunziato S, Adams DJ, Montini E (2013). Cancer gene discovery: exploiting insertional mutagenesis. Mol Cancer Res.

[70] March HN, Rust AG, Wright NA, ten Hoeve J, de Ridder J, Eldridge M, van der Weyden L, Berns A, Gadiot J, Uren A, Kemp R, Arends MJ, Wessels LF (2011). Insertional mutagenesis identifies multiple networks of cooperating genes driving intestinal tumorigenesis. Nat Genet.

[71] Bard-Chapeau EA, Nguyen AT, Rust AG, Sayadi A, Lee P, Chua BQ, New LS, de Jong J, Ward JM, Chin CK, Chew V, Toh HC, Abastado JP (2014). Transposon mutagenesis identifies genes driving hepatocellular carcinoma in a chronic hepatitis B mouse model. Nat Genet.

[72] Heckmann D, Maier P, Laufs S, Li L, Sleeman JP, Trunk MJ, Leupold JH, Wenz F, Zeller WJ, Fruehauf S, Allgayer H (2014). The disparate twins: a comparative study of CXCR4 and CXCR7 in SDF-1α-induced gene expression, invasion and chemosensitivity of colon cancer. Clin Cancer Res.

[73] Yang X, Wu K, Li S, Hu L, Han J, Zhu D, Tian X, Liu W, Tian Z, Zhong L, Yan M, Zhang C, Zhang Z (2017). MFAP5 and TNNC1: Potential markers for predicting occult cervical lymphatic metastasis and prognosis in early stage tongue cancer. Oncotarget.

[74] Monteith GR, McAndrew D, Faddy HM, Roberts-Thomson SJ (2007). Calcium and cancer: targeting Ca2+ transport. Nat Rev Cancer.

[75] Sergeev IN, Rhoten WB (1998). Regulation of intracellular calcium in human breast cancer cells. Endocrine.

[76] Abd-Rabou AA (2017). Calcium, a Cell Cycle Commander, Drives Colon Cancer Cell Diffpoptosis. Indian J Clin Biochem.

[77] Wang Y, He H, Li W, Phay J, Shen R, Yu L, Hancioglu B, de la Chapelle A (2017). MYH9 binds to lncRNA gene PTCSC2 and regulates FOXE1 in the 9q22 thyroid cancer risk locus. Proc Natl Acad Sci U S A.

[78] Baeriswyl V, Christofori G (2009). The angiogenic switch in carcinogenesis. Semin Cancer Biol.

[79] Bergers G, Benjamin LE (2003). Tumorigenesis and the angiogenic switch. Nat Rev Cancer.

[80] Dimova I, Popivanov G, Djonov V (2014). Angiogenesis in cancer -general pathways and their therapeutic implications. J BUON.

[81] Moses MA, Wiederschain D, Wu I, Fernandez CA, Ghazizadeh V, Lane WS, Flynn E, Sytkowski A, Tao T, Langer R (1999). Troponin I is present in human cartilage and inhibits angiogenesis. Proc Natl Acad Sci U S A.

[82] Fukumoto S, Sakaguchi T, You M, Xuan X, Fujisaki K (2006). Tick troponin I-like molecule is a potent inhibitor for angiogenesis. Microvasc Res.

[83] Xie Q, Yao S, Chen X, Xu L, Peng W, Zhang L, Zhang Q, Liang XF, Hong A (2012). A polypeptide from shark troponin I can inhibit angiogenesis and tumor growth. Mol Biol Rep.

[84] Feldman L, Rouleau C (2002). Troponin I inhibits capillary endothelial cell proliferation by interaction with the cell's bFGF receptor. Microvasc Res.

[85] Xiong G, Yang L, Wei Y, Wang S, Tian L, Lei S, Kan B, Mao Y (2007). Expression of the human fast-twitch skeletal muscle troponin I cDNA in a human ovarian carcinoma suppresses tumor growth. Sci China C Life Sci.

[86] Schmidt K, Hoffend J, Altmann A, Kiessling F, Strauss L, Koczan D, Mier W, Eisenhut M, Kinscherf R, Haberkorn U (2006). Troponin I overexpression inhibits tumor growth, perfusion, and vascularization of morris hepatoma. J Nucl Med.

[87] Haberkorn U, Hoffend J, Schmidt K, Altmann A, Bonaterra GA, Dimitrakopoulou-Strauss A, Strauss LG, Eisenhut M, Kinscherf R (2007). Changes in glucose metabolism and gene expression after transfer of anti-angiogenic genes in rat hepatoma. Eur J Nucl Med Mol Imaging.

[88] Van Eyk JE, Hodges RS (1988). The biological importance of each amino acid residue of the troponin I inhibitory sequence 104-115 in the interaction with troponin C and tropomyosin-actin. J Biol Chem.

[89] Talbot JA, Hodges RS (1981). Comparative studies on the inhibitory region of selected species of troponin-I. The use of synthetic peptide analogs to probe structure-function relationships. J Biol Chem.

[90] Dutour A, Rabinovich-Chable H, Kaletta C, Michaelis U, Fiorenza F, Sturtz F, Rigaud M (2004). Is troponin I gene therapy effective for osteosarcoma treatment? Study on a human-like orthotopic rat model. Anticancer Res.

[91] Kuroda T, Yasuda S, Nakashima H, Takada N, Matsuyama S, Kusakawa S, Umezawa A, Matsuyama A, Kawamata S, Sato Y (2017). Identification of a Gene Encoding Slow Skeletal Muscle Troponin T as a Novel Marker for Immortalization of Retinal Pigment Epithelial Cells. Sci Rep.

[92] Gupton SL, Anderson KL, Kole TP, Fischer RS, Ponti A, Hitchcock-DeGregori SE, Danuser G, Fowler VM, Wirtz D, Hanein D, Waterman-Storer CM (2005). Cell migration without a lamellipodium: translation of actin dynamics into cell movement mediated by tropomyosin. J Cell Biol.

[93] Lawrenson K, Pakzamir E, Liu B, Lee JM, Delgado MK, Duncan K, Gayther SA, Liu S, Roman L, Mhawech-Fauceglia P (2015). Molecular Analysis of Mixed Endometrioid and Serous Adenocarcinoma of the Endometrium. PLoS One.

[94] Ara MN, Hyodo M, Ohga N, Akiyama K, Hida K, Hida Y, Shinohara N, Harashima H (2014). Identification and expression of troponin T, a new marker on the surface of cultured tumor endothelial cells by aptamer ligand. Cancer Med.

[95] Gu X, Li B, Jiang M, Fang M, Ji J, Wang A, Wang M, Jiang X, Gao C (2015). RNA sequencing reveals differentially expressed genes as potential diagnostic and prognostic indicators of gallbladder carcinoma. Oncotarget.

[96] Uhlen M, Zhang C, Lee S, Sjöstedt E, Fagerberg L, Bidkhori G, Benfeitas R, Arif M, Liu Z, Edfors F, Sanli K, von Feilitzen K, Oksvold P (2017). A pathology atlas of the human cancer transcriptome. Science.

[97] Gross S, Rahal R, Stransky N, Lengauer C, Hoeflich KP (2015). Targeting cancer with kinase inhibitors. J Clin Invest.

[98] Zhao Y, Meng XM, Wei YJ, Zhao XW, Liu DQ, Cao HQ, Liew CC, Ding JF (2003). Cloning and characterization of a novel cardiac-specific kinase that interacts specifically with cardiac troponin I. J Mol Med (Berl).

[99] Wang X, Wang J, Su M, Wang C, Chen J, Wang H, Song L, Zou Y, Zhang L, Zhang Y, Hui R (2013). TNNI3K, a cardiac-specific kinase, promotes physiological cardiac hypertrophy in transgenic mice. PLoS One.

[100] Milano A, Lodder EM, Bezzina CR (2015). TNNI3K in cardiovascular disease and prospects for therapy. J Mol Cell Cardiol.

[101] Lawhorn BG, Philp J, Graves AP, Shewchuk L, Holt DA, Gatto GJ, Kallander LS (2016). GSK114: A selective inhibitor for elucidating the biological role of TNNI3K. Bioorg Med Chem Lett.

[102] EL Andaloussi S, Mäger I, Breakefield XO, Wood MJ (2013). Extracellular vesicles: biology and emerging therapeutic opportunities. Nat Rev Drug Discov.

[103] Raposo G, Stoorvogel W (2013). Extracellular vesicles: exosomes, microvesicles, and friends. J Cell Biol.

[104] Zhang X, Yuan X, Shi H, Wu L, Qian H, Xu W (2015). Exosomes in cancer: small particle, big player. J Hematol Oncol.

[105] Pan J, Ding M, Xu K, Yang C, Mao LJ (2017). Exosomes in diagnosis and therapy of prostate cancer. Oncotarget.

[106] Skog J, Würdinger T, van Rijn S, Meijer DH, Gainche L, Sena-Esteves M, Curry WT, Carter BS, Krichevsky AM, Breakefield XO (2008). Glioblastoma microvesicles transport RNA and proteins that promote tumour growth and provide diagnostic biomarkers. Nat Cell Biol.

[107] Siegel RL, Miller KD, Jemal A (2017). Cancer Statistics, 2017. CA Cancer J Clin.

[108] Florescu M, Cinteza M, Vinereanu D (2013). Chemotherapy-induced Cardiotoxicity. Maedica (Buchar).

[109] Pai VB, Nahata MC (2000). Cardiotoxicity of chemotherapeutic agents: incidence, treatment and prevention. Drug Saf.

[110] Keller T, Zeller T, Peetz D, Tzikas S, Roth A, Czyz E, Bickel C, Baldus S, Warnholtz A, Fröhlich M, Sinning CR, Eleftheriadis MS, Wild PS (2009). Sensitive troponin I assay in early diagnosis of acute myocardial infarction. N Engl J Med.

[111] Mair J, Genser N, Morandell D, Maier J, Mair P, Lechleitner P, Calzolari C, Larue C, Ambach E, Dienstl F, Pau B, Puschendorf B (1996). Cardiac troponin I in the diagnosis of myocardial injury and infarction. Clin Chim Acta.

[112] La Vecchia L, Mezzena G, Zanolla L, Paccanaro M, Varotto L, Bonanno C, Ometto R (2000). Cardiac troponin I as diagnostic and prognostic marker in severe heart failure. J Heart Lung Transplant.

[113] Henry NL, Hayes DF (2012). Cancer biomarkers. Mol Oncol.

[114] Franklin S, Zhang MJ, Chen H, Paulsson AK, Mitchell-Jordan SA, Li Y, Ping P, Vondriska TM (2011). Specialized compartments of cardiac nuclei exhibit distinct proteomic anatomy. Mol Cell Proteomics.

[115] Bergmann O, Zdunek S, Alkass K, Druid H, Bernard S, Frisén J (2011). Identification of cardiomyocyte nuclei and assessment of ploidy for the analysis of cell turnover. Exp Cell Res.

[116] Kajstura J, Urbanek K, Perl S, Hosoda T, Zheng H, Ogórek B, Ferreira-Martins J, Goichberg P, Rondon-Clavo C, Sanada F, D'Amario D, Rota M, Del Monte F (2010). Cardiomyogenesis in the adult human heart. Circ Res.

[117] Jeong EM, Wang X, Xu K, Hossain MM, Jin JP (2009). Nonmyofilament-associated troponin T fragments induce apoptosis. Am J Physiol Heart Circ Physiol.

[118] Delbono O (2011). Expression and regulation of excitation-contraction coupling proteins in aging skeletal muscle. Curr Aging Sci.

[119] Taylor J, Pereyra A, Zhang T, Messi ML, Wang ZM, Hereñú C, Kuan PF, Delbono O (2014). The Cavβ1a subunit regulates gene expression and suppresses myogenin in muscle progenitor cells. J Cell Biol.

[120] Zhang T, Pereyra AS, Wang ZM, Birbrair A, Reisz JA, Files DC, Purcell L, Feng X, Messi ML, Feng H, Chalovich J, Jin JP, Furdui C (2016). Calpain inhibition rescues troponin T3 fragmentation, increases Cav1.1, and enhances skeletal muscle force in aging sedentary mice. Aging Cell.

[121] Xu Z, Feng X, Dong J, Wang ZM, Lee J, Furdui C, Files DC, Beavers KM, Kritchevsky S, Milligan C, Jin JP, Delbono O, Zhang T (2017). Cardiac troponin T and fast skeletal muscle denervation in ageing. J Cachexia Sarcopenia Muscle.

[122] Harvey PA, Leinwand LA (2011). The cell biology of disease: cellular mechanisms of cardiomyopathy. J Cell Biol.

[123] Fatkin D, Graham RM (2002). Molecular mechanisms of inherited cardiomyopathies. Physiol Rev.

[124] Kimura A (2010). Molecular basis of hereditary cardiomyopathy: abnormalities in calcium sensitivity, stretch response, stress response and beyond. J Hum Genet.

[125] Pinto JR, Reynaldo DP, Parvatiyar MS, Dweck D, Liang J, Jones MA, Sorenson MM, Potter JD (2011). Strong cross-bridges potentiate the Ca2+ affinity changes produced by hypertrophic cardiomyopathy cardiac troponin C mutants in myofilaments: a fast kinetic approach. J Biol Chem.

[126] Pinto JR, Siegfried JD, Parvatiyar MS, Li D, Norton N, Jones MA, Liang J, Potter JD, Hershberger RE (2011). Functional characterization of TNNC1 rare variants identified in dilated cardiomyopathy. J Biol Chem.

[127] Baudenbacher F, Schober T, Pinto JR, Sidorov VY, Hilliard F, Solaro RJ, Potter JD, Knollmann BC (2008). Myofilament Ca2+ sensitization causes susceptibility to cardiac arrhythmia in mice. The Journal of clinical investigation.

[128] Hershberger RE, Pinto JR, Parks SB, Kushner JD, Li D, Ludwigsen S, Cowan J, Morales A, Parvatiyar MS, Potter JD (2009). Clinical and functional characterization of TNNT2 mutations identified in patients with dilated cardiomyopathy. Circulation Cardiovascular genetics.

[129] Martins AS, Parvatiyar MS, Feng HZ, Bos JM, Gonzalez-Martinez D, Vukmirovic M, Turna RS, Sanchez-Gonzalez MA, Badger CD, Zorio DA, Singh RK, Wang Y, Jin JP (2015). *In Vivo* Analysis of Troponin C Knock-In (A8V) Mice: Evidence that TNNC1 Is a Hypertrophic Cardiomyopathy Susceptibility Gene. Circ Cardiovasc Genet.

[130] Kawai M, Johnston JR, Karam T, Wang L, Singh RK, Pinto JR (2017). Myosin Rod Hypophosphorylation and CB Kinetics in Papillary Muscles from a TnC-A8V KI Mouse Model. Biophys J.

[131] Wen Y, Pinto JR, Gomes AV, Xu Y, Wang Y, Wang Y, Potter JD, Kerrick WG (2008). Functional Consequences of the Human Cardiac Troponin I Hypertrophic Cardiomyopathy Mutation R145G in Transgenic Mice. J Biol Chem.

[132] Veltri T, de Oliveira GA, Bienkiewicz EA, Palhano FL, Marques MA, Moraes AH, Silva JL, Sorenson MM, Pinto JR (2017). Amide hydrogens reveal a temperature-dependent structural transition that enhances site-II Ca2+-binding affinity in a C-domain mutant of cardiac troponin C.. Sci Rep.

[133] Veltri T, Landim-Vieira M, Parvatiyar MS, Gonzalez-Martinez D, Dieseldorff Jones KM, Michell CA, Dweck D, Landstrom AP, Chase PB, Pinto JR (2017). Hypertrophic Cardiomyopathy Cardiac Troponin C Mutations Differentially Affect Slow Skeletal and Cardiac Muscle Regulation. Front Physiol.

[134] Köhler J, Chen Y, Brenner B, Gordon AM, Kraft T, Martyn DA, Regnier M, Rivera AJ, Wang CK, Chase PB (2003). Familial hypertrophic cardiomyopathy mutations in troponin I (K183∆, G203S, K206Q) enhance filament sliding. Physiol Genomics.

[135] Brunet NM, Mihajlović G, Aledealat K, Wang F, Xiong P, von Molnár S, Chase PB (2012). Micromechanical thermal assays of Ca2+-regulated thin-filament function and modulation by hypertrophic cardiomyopathy mutants of human cardiac troponin. J Biomed Biotechnol.

[136] Brunet NM, Chase PB, Mihajlović G, Schoffstall B (2014). Ca2+-regulatory function of the inhibitory peptide region of cardiac troponin I is aided by the C-terminus of cardiac troponin T: Effects of familial hypertrophic cardiomyopathy mutations cTnI R145G and cTnT R278C, alone and in combination, on filament sliding. Arch Biochem Biophys.

[137] Phan NN, Wang CY, Lin YC (2014). The novel regulations of MEF2A, CAMKK2, CALM3, and TNNI3 in ventricular hypertrophy induced by arsenic exposure in rats. Toxicology.

[138] Cho DI, Kang WS, Hong MH, Kang HJ, Kim MR, Kim MC, Kim YS, Ahn Y (2017). The optimization of cell therapy by combinational application with apicidin-treated mesenchymal stem cells after myocardial infarction. Oncotarget.

[139] Leuschner F, Li J, Göser S, Reinhardt L, Ottl R, Bride P, Zehelein J, Pfitzer G, Remppis A, Giannitsis E, Katus HA, Kaya Z (2008). Absence of auto-antibodies against cardiac troponin I predicts improvement of left ventricular function after acute myocardial infarction. Eur Heart J.

[140] Kaya Z, Goser S, Buss SJ, Leuschner F, Ottl R, Li J, Volkers M, Zittrich S, Pfitzer G, Rose NR, Katus HA (2008). Identification of cardiac troponin I sequence motifs leading to heart failure by induction of myocardial inflammation and fibrosis. Circulation.

[141] Goser S, Andrassy M, Buss SJ, Leuschner F, Volz CH, Ottl R, Zittrich S, Blaudeck N, Hardt SE, Pfitzer G, Rose NR, Katus HA, Kaya Z (2006). Cardiac troponin I but not cardiac troponin T induces severe autoimmune inflammation in the myocardium. Circulation.

[142] Pinto JR, Muller-Delp J, Chase PB (2017). Will you still need me (Ca2+, TnT, and DHPR), will you still cleave me (calpain), when I'm 64?. Aging Cell.

[143] Dewenter M, von der Lieth A, Katus HA, Backs J (2017). Calcium Signaling and Transcriptional Regulation in Cardiomyocytes. Circ Res.

[144] Neidhardt L, Gasca S, Wertz K, Obermayr F, Worpenberg S, Lehrach H, Herrmann BG (2000). Large-scale screen for genes controlling mammalian embryogenesis, using high-throughput gene expression analysis in mouse embryos. Mech Dev.

[145] Schiaffino S, Reggiani C (2011). Fiber types in mammalian skeletal muscles. Physiol Rev.

[146] Fabregat A, Sidiropoulos K, Viteri G, Forner O, Marin-Garcia P, Arnau V, D'Eustachio P, Stein L, Hermjakob H (2017). Reactome pathway analysis: a high-performance in-memory approach. BMC Bioinformatics.

[147] Wu G, Haw R (2017). Functional Interaction Network Construction and Analysis for Disease Discovery. Methods Mol Biol.

[148] Huberts DH, van der Klei IJ (2010). Moonlighting proteins: an intriguing mode of multitasking. Biochim Biophys Acta.

[149] Jeffery CJ (2003). Moonlighting proteins: old proteins learning new tricks. Trends Genet.

[150] Vassylyev DG, Takeda S, Wakatsuki S, Maeda K, Maéda Y (1998). Crystal structure of troponin C in complex with troponin I fragment at 2.3-Å resolution. Proc Natl Acad Sci U S A.

[151] Na I, Kong MJ, Straight S, Pinto JR, Uversky VN (2016). Troponins, intrinsic disorder, and cardiomyopathy. Biol Chem.

[152] Pinto JR, Gomes AV, Jones MA, Liang J, Nguyen S, Miller T, Parvatiyar MS, Potter JD (2012). The functional properties of human slow skeletal troponin T isoforms in cardiac muscle regulation. J Biol Chem.

[153] Kowlessur D, Tobacman LS (2010). Troponin regulatory function and dynamics revealed by H/D exchange-mass spectrometry. J Biol Chem.

[154] Szilágyi A, Skolnick J (2006). Efficient prediction of nucleic acid binding function from low-resolution protein structures. J Mol Biol.

