# Supplementary material for: Troponin through the looking-glass: emerging roles beyond regulation of striated muscle contraction
Source: Oncotarget. 2017 Dec 4;9(1):1461–82. doi: 10.18632/oncotarget.22879 (PMC5787451; doi:10.18632/oncotarget.22879)
Supplement: Supplementary file 1 [file oncotarget-09-1461-s001.pdf]

Troponin through the looking-glass: emerging roles beyond regulation of striated muscle contraction

SUPPLEMENTARY MATERIALS

Supplementary Table 1: Somatic mutations in *TNNC1* gene associated with cancer types

CDS Mutation	AA Mutation	Mutation ID (COSM)	Count	Type
c.10A>C	p.I4L	5865006	1	Substitution - Missense
c.20C>A	p.A7D	6275095	1	Substitution - Missense
c.45G>T	p.E15D	139460	1	Substitution - Missense
c.60C>A	p.F20L	5465926	2	Substitution - Missense
c.73G>A	p.D25N	3596077	1	Substitution - Missense
c.91G>A	p.A31T	252882	1	Substitution - Missense
c.110G>A	p.S37N	1229955	1	Substitution - Missense
c.146G>T	p.G49V	5497715	1	Substitution - Missense
c.166G>A	p.E56K	5898834	1	Substitution - Missense
c.202G>A	p.G68S	2853594	1	Substitution - Missense
c.248G>A	p.R83Q	1229956	1	Substitution - Missense
c.262G>A	p.D88N	1047014	3	Substitution - Missense
c.271G>T	p.G91W	1047013	1	Substitution - Missense
c.329G>T	p.G110V	4119079	1	Substitution - Missense
c.418G>A	p.G140R	1692816	2	Substitution - Missense
c.419G>A	p.G140E	3596072	4	Substitution - Missense
c.421G>A	p.D141N	5934835	1	Substitution - Missense
c.439C>T	p.R147C	731105	2	Substitution - Missense
c.440G>A	p.R147H	1268237	2	Substitution - Missense
c.445G>A	p.D149N	1424489	1	Substitution - Missense

Data were exported as CSV files from the online Catalogue of Somatic Mutations in Cancer (COSMIC) database (http://cancer.sanger.ac.uk/cosmic) on August 12th, 2017. For brevity, synonymous mutations were omitted. CDS = coding DNA sequencing; AA = amino acid; COSM = COSMIC ID.

Supplementary Table 2: Somatic mutations in *TNNC2* gene associated with cancer types

CDS Mutation	AA Mutation	Mutation ID (COSM)	Count	Type
c.5C>T	p.T2M	3405150	1	Substitution - Missense
c.16G>A	p.A6T	3547097	1	Substitution - Missense
c.26G>T	p.R9M	4735028	1	Substitution - Missense
c.32A>C	p.Y11S	3293761	1	Substitution - Missense
c.64G>A	p.A22T	6033309	1	Substitution - Missense
c.74A>C	p.D25A	4735027	1	Substitution - Missense
c.86C>A	p.A29D	4134590	1	Substitution - Missense
c.88G>C	p.D30H	4427289	1	Substitution - Missense
c.113_114insA	p.E39fs* 27	1027290	1	Insertion - Frameshift
c.125C>T	p.T42M	3693529	3	Substitution - Missense
c.131T>A	p.M44K	365214	1	Substitution - Missense
c.134G>C	p.R45T	3547096	1	Substitution - Missense
c.166C>A	p.L56M	1318832	1	Substitution - Missense
c.171C>A	p.D57E	6314551	1	Substitution - Missense
c.180C>G	p.I60M	376770	1	Substitution - Missense
c.193G>A	p.E65K	6093532	1	Substitution - Missense
c.202A>C	p.S68R	4924870	1	Substitution - Missense
c.220G>C	p.E74Q	3841135	1	Substitution - Missense
c.227T>C	p.F76S	1027289	1	Substitution - Missense
c.273G>C	p.K91N	443911	1	Substitution - Missense
c.304A>G	p.I102V	5557536	1	Substitution - Missense
c.319G>T	p.A107S	3713281	2	Substitution - Missense
c.365C>T	p.A122V	5857984	1	Substitution - Missense
c.397G>A	p.E133K	3547095	1	Substitution - Missense
c.408G>T	p.M136I	97846	2	Substitution - Missense
c.409A>C	p.K137Q	4400718	1	Substitution - Missense
c.415G>C	p.G139R	5790019	1	Substitution - Missense
c.471G>T	p.E157D	1326713	1	Substitution - Missense
c.473G>C	p.G158A	5789710	1	Substitution - Missense
c.472G>A	p.G158S	5454842	1	Substitution - Missense
c.475G>A	p.V159M	4656651	1	Substitution - Missense
c.474delC	p.V159fs* >2	5018450	1	Deletion – Frameshift

Data were exported as CSV files from the online Catalogue of Somatic Mutations in Cancer (COSMIC) database (http://cancer.sanger.ac.uk/cosmic) on August 12th, 2017. For brevity, synonymous mutations were omitted. CDS = coding DNA sequencing; AA = amino acid; COSM = COSMIC ID.

Supplementary Table 3: Somatic mutations in *TNNI1* gene associated with cancer types

CDS Mutation	AA Mutation	Mutation ID (COSM)	Count	Type
c.16A>G	p.R6G	6302416	1	Substitution - Missense
c.25A>T	p.K9*	6392652	1	Substitution - Nonsense
c.47T>C	p.L16P	463750	1	Substitution - Missense
c.71C>T	p.A24V	1229957	1	Substitution - Missense
c.83A>C	p.E28A	5845094	2	Substitution - Missense
c.103G>A	p.E35K	1500466	4	Substitution - Missense
c.119A>G	p.E40G	1668361	4	Substitution - Missense
c.127C>T	p.R43C	5146960	1	Substitution - Missense
c.128G>A	p.R43H	255848	2	Substitution - Missense
c.136G>A	p.A46T	2212422	1	Substitution - Missense
c.155T>C	p.L52P	6372330	1	Substitution - Missense
c.182C>T	p.A61V	6307360	1	Substitution - Missense
c.199C>T	p.R67W	1268238	1	Substitution - Missense
c.200G>A	p.R67Q	1648456	2	Substitution - Missense
c.210C>A	p.H70Q	6123650	1	Substitution - Missense
c.222G>T	p.E74D	4026940	1	Substitution - Missense
c.224T>C	p.V75A	5845093	1	Substitution - Missense
c.250G>A	p.E84K	5441535	1	Substitution - Missense
c.269A>G	p.N90S	3966175	1	Substitution - Missense
c.286G>A	p.D96N	3864097	1	Substitution - Missense
c.329G>A	p.R110H	267608	1	Substitution - Missense
c.341G>A	p.R114H	1290087	1	Substitution - Missense
c.344G>A	p.R115Q	5121910	1	Substitution - Missense
c.349C>T	p.R117C	5889622	1	Substitution - Missense
c.361G>A	p.D121N	5698114	1	Substitution - Missense
c.367A>G	p.M123V	902193	1	Substitution - Missense
c.373C>T	p.R125W	1295685	1	Substitution - Missense
c.408G>A	p.M136I	227985	1	Substitution - Missense
c.433G>A	p.V145M	6367320	1	Substitution - Missense
c.460C>T	p.R154W	5091544	1	Substitution - Missense
c.468_469GG>AA	p.E157K	4447733	1	Substitution - Missense
c.489G>C	p.K163N	355950	1	Substitution - Missense
c.520C>T	p.R174W	1668360	2	Substitution - Missense
c.521G>A	p.R174Q	295577	1	Substitution – Missense

Data were exported as CSV files from the online Catalogue of Somatic Mutations in Cancer (COSMIC) database (http://cancer.sanger.ac.uk/cosmic) on August 12th, 2017. For brevity, synonymous mutations were omitted. CDS = coding DNA sequencing; AA = amino acid; COSM = COSMIC ID.

Supplementary Table 4: Somatic mutations in *TNNI2* gene associated with cancer types

CDS Mutation	AA Mutation	Mutation ID (COSM)	Count	Type
c.1A>T	p.M1L	4922370	1	Substitution - Missense
c.38C>A	p.A13D	6131860	1	Substitution - Missense
c.41G>A	p.R14H	4031922	1	Substitution - Missense
c.45G>C	p.R15S	323911	1	Substitution - Missense
c.57G>T	p.K19N	241974	1	Substitution - Missense
c.71A>G	p.Q24R	925672	1	Substitution - Missense
c.74T>G	p.I25R	323910	1	Substitution - Missense
c.88C>A	p.L30M	3415743	2	Substitution - Missense
c.89T>A	p.L30Q	686993	1	Substitution - Missense
c.101A>T	p.E34V	4930971	1	Substitution - Missense
c.110G>T	p.R37L	342102	1	Substitution - Missense
c.152C>T	p.P51L	4031923	1	Substitution - Missense
c.178G>A	p.E60K	415234	1	Substitution - Missense
c.182T>A	p.V61E	6068074	1	Substitution - Missense
c.206A>G	p.H69R	925673	1	Substitution - Missense
c.208G>A	p.A70T	1353016	1	Substitution - Missense
c.213G>T	p.K71N	6400913	1	Substitution - Missense
c.217G>A	p.D73N	925674	2	Substitution - Missense
c.223G>A	p.A75T	4031924	1	Substitution - Missense
c.241G>A	p.D81N	5625068	1	Substitution - Missense
c.246G>A	p.M82I	3446017	1	Substitution - Missense
c.251T>C	p.V84A	4646338	1	Substitution - Missense
c.270C>A	p.S90R	6398803	1	Substitution - Missense
c.274G>A	p.E92K	925675	1	Substitution - Missense
c.278T>C	p.L93P	4735029	1	Substitution - Missense
c.337C>T	p.R113W	4031925	2	Substitution - Missense
c.338G>A	p.R113Q	5469559	1	Substitution - Missense
c.338G>C	p.R113P	395439	1	Substitution - Missense
c.347G>C	p.R116P	337246	1	Substitution - Missense
c.390G>T	p.K130N	1353017	1	Substitution - Missense
c.395A>C	p.K132T	2092799	1	Substitution - Missense
c.406G>A	p.D136N	3446019	1	Substitution - Missense
c.420C>G	p.N140K	4031926	1	Substitution - Missense
c.446C>T	p.T149I	6379152	1	Substitution - Missense
c.451A>T	p.K151*	6068073	1	Substitution - Nonsense
c.467G>A	p.R156Q	1353018	1	Substitution - Missense
c.470A>G	p.D157G	6392787	1	Substitution - Missense
c.483G>A	p.W161*	1722817	2	Substitution - Nonsense
c.520C>T	p.R174W	4031927	1	Substitution - Missense
c.541G>C	p.E181Q	4972580	2	Substitution - Missense

Data were exported as CSV files from the online Catalogue of Somatic Mutations in Cancer (COSMIC) database (http://cancer.sanger.ac.uk/cosmic) on August 12th, 2017. For brevity, synonymous mutations were omitted. CDS = coding DNA sequencing; AA = amino acid; COSM = COSMIC ID.

Supplementary Table 5: Somatic mutations in *TNNI3* gene associated with cancer types

See Supplementary File 1

Supplementary Table 6: Somatic mutations in *TNNT1* gene associated with cancer types

See Supplementary File 1

Supplementary Table 7: Somatic mutations in *TNNT2* gene associated with cancer types

See Supplementary File 1

Supplementary Table 8: Somatic mutations in *TNNT3* gene associated with cancer types

See Supplementary File 1
