# Supplementary material for: Troponin through the looking-glass: emerging roles beyond regulation of striated muscle contraction
Source: Oncotarget. 2017 Dec 4;9(1):1461–82. doi: 10.18632/oncotarget.22879 (PMC5787451; doi:10.18632/oncotarget.22879)
Supplement: Supplementary file 2 [file oncotarget-09-1461-s002.docx]

**Supplementary Table 5. Somatic mutations in *TNNI3* gene associated with cancer types**

| CDS Mutation | AA Mutation | Mutation ID (COSM) | Count | Type |
| --- | --- | --- | --- | --- |
| c.20_24delATGCG | p.D7fs*2 | 5546856 | 1 | Deletion - Frameshift |
| c.37C>T | p.R13C | 4881874 | 1 | Substitution - Missense |
| c.38G>A | p.R13H | 2886583 | 1 | Substitution - Missense |
| c.40C>T | p.P14S | 3539352 | 1 | Substitution - Missense |
| c.41C>A | p.P14H | 4735034 | 1 | Substitution - Missense |
| c.56T>C | p.I19T | 3835804 | 1 | Substitution - Missense |
| c.62G>T | p.R21L | 6386933 | 1 | Substitution - Missense |
| c.67T>C | p.S23P | 4735033 | 1 | Substitution - Missense |
| c.71C>T | p.S24F | 6374475 | 1 | Substitution - Missense |
| c.80G>A | p.R27H | 4735032 | 1 | Substitution - Missense |
| c.85T>C | p.Y29H | 5165705 | 1 | Substitution - Missense |
| c.95A>C | p.E32A | 395732 | 1 | Substitution - Missense |
| c.102C>G | p.H34Q | 84550 | 1 | Substitution - Missense |
| c.110A>C | p.K37T | 4638255 | 1 | Substitution - Missense |
| c.230G>A | p.S77N | 418024 | 1 | Substitution - Missense |
| c.243G>C | p.Q81H | 3770148 | 1 | Substitution - Missense |
| c.283G>A | p.D95N | 4426934 | 1 | Substitution - Missense |
| c.292C>T | p.R98* | 4581429 | 1 | Substitution - Nonsense |
| c.293G>C | p.R98P | 6152260 | 1 | Substitution - Missense |
| c.301C>T | p.H101Y | 6086043 | 1 | Substitution - Missense |
| c.308G>A | p.R103H | 267609 | 1 | Substitution - Missense |
| c.308G>T | p.R103L | 713518 | 1 | Substitution - Missense |
| c.325G>T | p.E109* | 6152261 | 1 | Substitution - Nonsense |
| c.330G>C | p.E110D | 349666 | 1 | Substitution - Missense |
| c.332G>T | p.R111I | 4735031 | 1 | Substitution - Missense |
| c.337G>A | p.D113N | 5856775 | 1 | Substitution - Missense |
| c.350A>G | p.K117R | 440340 | 1 | Substitution - Missense |
| c.370G>A | p.E124K | 3893519 | 1 | Substitution - Missense |
| c.388C>T | p.Q130* | 713519 | 1 | Substitution - Nonsense |
| c.407G>A | p.R136Q | 4981186 | 2 | Substitution - Missense |
| c.422G>A | p.R141Q | 5056941 | 1 | Substitution - Missense |
| c.433C>T | p.R145W | 475295 | 1 | Substitution - Missense |
| c.468G>T | p.Q156H | 1190154 | 1 | Substitution - Missense |
| c.470C>T | p.A157V | 6388939 | 1 | Substitution - Missense |
| c.485G>A | p.R162Q | 6212838 | 1 | Substitution - Missense |
| c.488C>A | p.A163D | 1001403 | 2 | Substitution - Missense |
| c.509G>A | p.R170Q | 4429422 | 1 | Substitution - Missense |
| c.522G>T | p.K174N | 1001402 | 1 | Substitution - Missense |
| c.556C>T | p.R186W | 1001401 | 1 | Substitution - Missense |
| c.560A>G | p.E187G | 1001400 | 1 | Substitution - Missense |
| c.574C>T | p.R192C | 1001399 | 1 | Substitution - Missense |
| c.575G>A | p.R192H | 5183237 | 1 | Substitution - Missense |
| c.584T>C | p.I195T | 1612836 | 2 | Substitution - Missense |
| c.610C>T | p.R204C | 5440902 | 1 | Substitution - Missense |
| c.611G>T | p.R204L | 5699804 | 1 | Substitution - Missense |
| c.625G>A | p.E209K | 3835802 | 1 | Substitution - Missense |

Data were exported as CSV files from the online Catalogue of Somatic Mutations in Cancer (COSMIC) database (<http://cancer.sanger.ac.uk/cosmic>) on August 12^th^, 2017. For brevity, synonymous mutations were omitted. CDS = coding DNA sequencing; AA = amino acid; COSM = COSMIC ID

**Supplementary Table 6. Somatic mutations in *TNNT1* gene associated with cancer types**

| CDS Mutation | AA Mutation | Mutation ID (COSM) | Count | Type |
| --- | --- | --- | --- | --- |
| c.20A>G | p.Q7R | 1001398 | 1 | Substitution - Missense |
| c.39G>T | p.Q13H | 6369048 | 1 | Substitution - Missense |
| c.56C>T | p.A19V | 6363188 | 1 | Substitution - Missense |
| c.73G>A | p.A25T | 4557731 | 1 | Substitution - Missense |
| c.116G>A | p.R39H | 1001397 | 1 | Substitution - Missense |
| c.115C>T | p.R39C | 4081496 | 3 | Substitution - Missense |
| c.119C>A | p.P40H | 1196496 | 2 | Substitution - Missense |
| c.129C>A | p.S43R | 6298363 | 1 | Substitution - Missense |
| c.149T>C | p.L50S | 4735037 | 1 | Substitution - Missense |
| c.149T>A | p.L50* | 6152264 | 1 | Substitution - Nonsense |
| c.169G>C | p.E57Q | 1305119 | 1 | Substitution - Missense |
| c.197T>A | p.I66N | 1229960 | 1 | Substitution - Missense |
| c.202C>T | p.R68C | 6446430 | 1 | Substitution - Missense |
| c.229G>A | p.E77K | 5856774 | 2 | Substitution - Missense |
| c.238A>G | p.T80A | 6374919 | 1 | Substitution - Missense |
| c.270G>T | p.K90N | 4081495 | 1 | Substitution - Missense |
| c.280G>C | p.E94Q | 3713168 | 2 | Substitution - Missense |
| c.314G>A | p.R105Q | 3389385 | 1 | Substitution - Missense |
| c.313C>T | p.R105W | 713522 | 1 | Substitution - Missense |
| c.320G>T | p.R107L | 3960523 | 1 | Substitution - Missense |
| c.320G>A | p.R107Q | 1305118 | 1 | Substitution - Missense |
| c.334G>A | p.E112K | 3423176 | 1 | Substitution - Missense |
| c.361G>A | p.E121K | 3893518 | 1 | Substitution - Missense |
| c.364C>T | p.R122C | 5343438 | 1 | Substitution - Missense |
| c.364C>G | p.R122G | 378276 | 1 | Substitution - Missense |
| c.367G>A | p.E123K | 3893517 | 2 | Substitution - Missense |
| c.386C>T | p.A129V | 6328261 | 1 | Substitution - Missense |
| c.406G>A | p.E136K | 3539350 | 2 | Substitution - Missense |
| c.415G>A | p.E139K | 3797540 | 1 | Substitution - Missense |
| c.419C>G | p.A140G | 6152265 | 1 | Substitution - Missense |
| c.423G>T | p.K141N | 1001395 | 1 | Substitution - Missense |
| c.425A>T | p.K142M | 5623885 | 1 | Substitution - Missense |
| c.428G>A | p.R143Q | 3971265 | 1 | Substitution - Missense |
| c.427C>G | p.R143G | 4950755 | 2 | Substitution - Missense |
| c.447G>C | p.K149N | 1001394 | 1 | Substitution - Missense |
| c.453G>T | p.K151N | 5464758 | 1 | Substitution - Missense |
| c.487G>A | p.G163S | 2886554 | 2 | Substitution - Missense |
| c.492C>A | p.Y164* | 4608793 | 1 | Substitution - Nonsense |
| c.513G>C | p.K171N | 3960522 | 1 | Substitution - Missense |
| c.514C>T | p.R172C | 2886552 | 2 | Substitution - Missense |
| c.524G>A | p.R175Q | 3404633 | 3 | Substitution - Missense |
| c.523C>T | p.R175W | 6367621 | 1 | Substitution - Missense |
| c.551G>A | p.R184H | 5499387 | 1 | Substitution - Missense |
| c.562G>A | p.E188K | 4081494 | 1 | Substitution - Missense |
| c.586G>C | p.D196H | 73036 | 1 | Substitution - Missense |
| c.596G>T | p.G199V | 5343437 | 1 | Substitution - Missense |
| c.598delG | p.E200fs*41 | 2886545 | 1 | Deletion - Frameshift |
| c.611G>A | p.R204Q | 2151163 | 2 | Substitution - Missense |
| c.620C>G | p.S207C | 475294 | 1 | Substitution - Missense |
| c.634C>T | p.P212S | 5543549 | 1 | Substitution - Missense |
| c.655G>T | p.A219S | 5653572 | 1 | Substitution - Missense |
| c.673G>T | p.E225* | 395731 | 1 | Substitution - Nonsense |
| c.736C>A | p.Q246K | 5703335 | 1 | Substitution - Missense |
| c.751A>T | p.I251F | 3835800 | 1 | Substitution - Missense |
| c.753C>G | p.I251M | 3835799 | 1 | Substitution - Missense |
| c.769C>A | p.R257S | 6215462 | 1 | Substitution - Missense |
| c.770G>A | p.R257H | 475292 | 1 | Substitution - Missense |
| c.781G>A | p.A261T | 4642365 | 1 | Substitution - Missense |
| c.790T>G | p.F264V | 4081493 | 1 | Substitution - Missense |
| c.793C>A | p.R265S | 5697041 | 1 | Substitution - Missense |
| c.827G>A | p.R276H | 4081492 | 1 | Substitution - Missense |

Data were exported as CSV files from the online Catalogue of Somatic Mutations in Cancer (COSMIC) database (<http://cancer.sanger.ac.uk/cosmic>) on August 12^th^, 2017. For brevity, synonymous mutations were omitted. CDS = coding DNA sequencing; AA = amino acid; COSM = COSMIC ID

**Supplementary Table 7. Somatic mutations in *TNNT2* gene associated with cancer types**

| CDS Mutation | AA Mutation | Mutation ID (COSM) | Count | Type |
| --- | --- | --- | --- | --- |
| c.13G>T | p.E5* | 902184 | 1 | Substitution - Nonsense |
| c.34G>A | p.E12K | 5845090 | 1 | Substitution - Missense |
| c.37G>A | p.E13K | 5582814 | 1 | Substitution - Missense |
| c.43G>A | p.E15K | 5379203 | 1 | Substitution - Missense |
| c.69G>T | p.E23D | 1229962 | 1 | Substitution - Missense |
| c.73G>A | p.E25K | 5540616 | 1 | Substitution - Missense |
| c.101C>T | p.A34V | 4026929 | 1 | Substitution - Missense |
| c.116A>T | p.E39V | 6123658 | 1 | Substitution - Missense |
| c.125G>T | p.R42M | 2212357 | 1 | Substitution - Missense |
| c.160G>T | p.E54* | 902181 | 1 | Substitution - Nonsense |
| c.181G>A | p.E61K | 3481543 | 1 | Substitution - Missense |
| c.206C>T | p.S69L | 3481540 | 1 | Substitution - Missense |
| c.220T>G | p.L74V | 4764352 | 1 | Substitution - Missense |
| c.229C>T | p.P77S | 4826135 | 1 | Substitution - Missense |
| c.234G>T | p.K78N | 4979661 | 2 | Substitution - Missense |
| c.241G>A | p.D81N | 6349717 | 1 | Substitution - Missense |
| c.254T>A | p.V85E | 1337835 | 1 | Substitution - Missense |
| c.253_254insAG | p.V85fs*16 | 273757 | 1 | Insertion - Frameshift |
| c.259T>C | p.F87L | 4143205 | 1 | Substitution - Missense |
| c.261T>A | p.F87L | 4143202 | 1 | Substitution - Missense |
| c.274C>T | p.R92W | 4026926 | 1 | Substitution - Missense |
| c.280C>T | p.R94C | 5469837 | 1 | Substitution - Missense |
| c.281G>A | p.R94H | 902180 | 2 | Substitution - Missense |
| c.286G>C | p.E96Q | 3943318 | 1 | Substitution - Missense |
| c.286G>T | p.E96* | 1337833 | 1 | Substitution - Nonsense |
| c.311C>T | p.A104V | 4735047 | 1 | Substitution - Missense |
| c.319G>T | p.E107* | 6428517 | 1 | Substitution - Nonsense |
| c.361G>A | p.V121I | 4167680 | 1 | Substitution - Missense |
| c.373G>A | p.D125N | 3481534 | 1 | Substitution - Missense |
| c.386G>C | p.R129T | 3385613 | 2 | Substitution - Missense |
| c.389G>T | p.R130L | 678181 | 1 | Substitution - Missense |
| c.415C>A | p.R139S | 5807623 | 2 | Substitution - Missense |
| c.420delC | p.R141fs*41 | 1683819 | 1 | Deletion - Frameshift |
| c.422G>A | p.R141Q | 1229961 | 1 | Substitution - Missense |
| c.442C>T | p.R148W | 458314 | 2 | Substitution - Missense |
| c.447G>T | p.Q149H | 1229963 | 1 | Substitution - Missense |
| c.458C>G | p.A153G | 6060554 | 1 | Substitution - Missense |
| c.472C>T | p.R158* | 4735044 | 1 | Substitution - Nonsense |
| c.473G>T | p.R158L | 214070 | 1 | Substitution - Missense |
| c.490A>G | p.N164D | 4735041 | 1 | Substitution - Missense |
| c.492C>G | p.N164K | 4735038 | 1 | Substitution - Missense |
| c.494G>A | p.R165K | 6056088 | 1 | Substitution - Missense |
| c.502G>A | p.A168T | 902179 | 1 | Substitution - Missense |
| c.508G>C | p.D170H | 5966329 | 1 | Substitution - Missense |
| c.514G>A | p.A172T | 4958867 | 2 | Substitution - Missense |
| c.541A>G | p.M181V | 678182 | 1 | Substitution - Missense |
| c.565C>T | p.Q189* | 3864090 | 1 | Substitution - Nonsense |
| c.566A>T | p.Q189L | 2155066 | 1 | Substitution - Missense |
| c.577C>T | p.R193W | 902178 | 1 | Substitution - Missense |
| c.589A>G | p.K197E | 902177 | 1 | Substitution - Missense |
| c.604C>T | p.R202W | 1689605 | 1 | Substitution - Missense |
| c.607_608insA | p.K205fs*7 | 1190992 | 1 | Insertion - Frameshift |
| c.620_622delAGA | p.K207delK | 1337830 | 1 | Deletion - In frame |
| c.670G>C | p.D224H | 165041 | 1 | Substitution - Missense |
| c.729G>T | p.E243D | 902176 | 1 | Substitution - Missense |
| c.744G>T | p.Q248H | 6123664 | 1 | Substitution - Missense |
| c.785G>A | p.R262Q | 902175 | 2 | Substitution - Missense |
| c.807G>C | p.Q269H | 251175 | 2 | Substitution - Missense |
| c.824G>A | p.R275H | 902174 | 1 | Substitution - Missense |
| c.847C>T | p.R283C | 5456239 | 1 | Substitution - Missense |

Data were exported as CSV files from the online Catalogue of Somatic Mutations in Cancer (COSMIC) database (<http://cancer.sanger.ac.uk/cosmic>) on August 12^th^, 2017. For brevity, synonymous mutations were omitted. CDS = coding DNA sequencing; AA = amino acid; COSM = COSMIC ID

**Supplementary Table 8. Somatic mutations in *TNNT3* gene associated with cancer types**

| CDS Mutation | AA Mutation | Mutation ID (COSM) | Count | Type |
| --- | --- | --- | --- | --- |
| c.15A>C | p.E5D | 925826 | 1 | Substitution - Missense |
| c.39C>A | p.Y13* | 1235754 | 1 | Substitution - Nonsense |
| c.40G>A | p.E14K | 1703333 | 1 | Substitution - Missense |
| c.43G>A | p.E15K | 5586822 | 1 | Substitution - Missense |
| c.40_42delGAA | p.E18delE | 2093376 | 1 | Deletion - In frame |
| c.60G>T | p.Q20H | 6398072 | 1 | Substitution - Missense |
| c.61G>A | p.E21K | 4554389 | 1 | Substitution - Missense |
| c.86C>A | p.T29N | 686957 | 1 | Substitution - Missense |
| c.88G>A | p.A30T | 3687224 | 1 | Substitution - Missense |
| c.89C>T | p.A30V | 3769269 | 1 | Substitution - Missense |
| c.91G>A | p.E31K | 3446168 | 1 | Substitution - Missense |
| c.96G>C | p.E32D | 4165740 | 1 | Substitution - Missense |
| c.97G>A | p.D33N | 4165741 | 1 | Substitution - Missense |
| c.103G>A | p.E35K | 3446169 | 1 | Substitution - Missense |
| c.109G>A | p.E37K | 224977 | 1 | Substitution - Missense |
| c.119G>T | p.R40I | 1353115 | 1 | Substitution - Missense |
| c.136C>T | p.P46S | 3446170 | 1 | Substitution - Missense |
| c.152G>A | p.G51E | 5422871 | 1 | Substitution - Missense |
| c.155A>T | p.E52V | 6433434 | 1 | Substitution - Missense |
| c.157A>C | p.K53Q | 5935807 | 1 | Substitution - Missense |
| c.178C>A | p.Q60K | 4808390 | 1 | Substitution - Missense |
| c.188G>A | p.R63H | 428812 | 2 | Substitution - Missense |
| c.253G>A | p.E85K | 1240475 | 2 | Substitution - Missense |
| c.250_251insAGG | p.E88_L89insE | 5222921 | 1 | Insertion - In frame |
| c.280G>A | p.E94K | 229186 | 1 | Substitution - Missense |
| c.291G>T | p.E97D | 925830 | 1 | Substitution - Missense |
| c.296G>A | p.R99H | 3849500 | 2 | Substitution - Missense |
| c.299G>A | p.R100H | 4735051 | 1 | Substitution - Missense |
| c.301G>A | p.A101T | 466660 | 1 | Substitution - Missense |
| c.308G>A | p.R103K | 247934 | 1 | Substitution - Missense |
| c.321G>T | p.Q107H | 5690038 | 1 | Substitution - Missense |
| c.350G>A | p.R117H | 4735055 | 2 | Substitution - Missense |
| c.365C>T | p.A122V | 4735059 | 1 | Substitution - Missense |
| c.383G>A | p.R128K | 1703335 | 1 | Substitution - Missense |
| c.384G>T | p.R128S | 5779273 | 1 | Substitution - Missense |
| c.385G>A | p.E129K | 1703337 | 1 | Substitution - Missense |
| c.384_386delGGA | p.E131delE | 4746494 | 1 | Deletion - In frame |
| c.397G>A | p.A133T | 6461701 | 1 | Substitution - Missense |
| c.429G>T | p.K143N | 925831 | 1 | Substitution - Missense |
| c.443C>T | p.S148F | 4928436 | 1 | Substitution - Missense |
| c.446C>T | p.S149F | 3446176 | 1 | Substitution - Missense |
| c.451G>A | p.G151R | 5916125 | 1 | Substitution - Missense |
| c.475G>C | p.A159P | 687785 | 1 | Substitution - Missense |
| c.514C>T | p.R172W | 4735063 | 1 | Substitution - Missense |
| c.522G>C | p.M174I | 229773 | 1 | Substitution - Missense |
| c.522_524delGAA | p.K177delK | 2093412 | 2 | Deletion - In frame |
| c.530A>T | p.K177M | 6201769 | 1 | Substitution - Missense |
| c.541G>C | p.E181Q | 6132297 | 1 | Substitution - Missense |
| c.553C>A | p.P185T | 5818419 | 2 | Substitution - Missense |
| c.604G>A | p.E202K | 5593510 | 1 | Substitution - Missense |
| c.660G>C | p.K220N | 3368330 | 1 | Substitution - Missense |
| c.667C>T | p.R223C | 88693 | 4 | Substitution - Missense |
| c.701G>A | p.R234H | 4735067 | 1 | Substitution - Missense |
| c.709C>A | p.Q237K | 5882047 | 1 | Substitution - Missense |
| c.730A>T | p.K244* | 6279107 | 1 | Substitution - Nonsense |
| c.733G>C | p.A245P | 687783 | 1 | Substitution - Missense |
| c.746C>T | p.A249V | 4386572 | 1 | Substitution - Missense |
| c.760G>A | p.G254S | 925835 | 1 | Substitution - Missense |
| c.771G>A | p.W257* | 5838311 | 2 | Substitution - Nonsense |

Data were exported as CSV files from the online Catalogue of Somatic Mutations in Cancer (COSMIC) database (<http://cancer.sanger.ac.uk/cosmic>) on August 12^th^, 2017. For brevity, synonymous mutations were omitted. CDS = coding DNA sequencing; AA = amino acid; COSM = COSMIC ID.
